# Metabolic reprogramming of efferocytosis in the tumour microenvironment: From apoptotic‐cell clearance to therapeutic targeting

**DOI:** 10.1002/ctm2.70601

**Published:** 2026-01-28

**Authors:** Qianlu Yang, Jie Yan, Qianxi Yang

**Affiliations:** ^1^ Department of Breast Surgery of the Third Affiliated Hospital of Kunming Medical University Yunnan Cancer Hospital, Peking University Cancer Hospital Yunnan Kunming China; ^2^ Yunnan Eye Institute & Key Laboratory of Yunnan Province, Yunnan Eye Disease Clinical Medical Center Affiliated Hospital of Yunnan University, Yunnan University Kunming China

**Keywords:** Cancer immunotherapy, Efferocytosis, Immunometabolism, Metabolic reprogramming, Tumour microenvironment (TME)

## Abstract

**Background:**

Efferocytosis is a critical physiological process in which phagocytes clear apoptotic cells to maintain tissue homeostasis. However, within the tumour microenvironment (TME), this process is systematically hijacked by tumour cells, transforming it into a key pathological mechanism that drives immunosuppression, tumour progression and therapeutic resistance.

**Key findings:**

This review systematically elucidates the central role of metabolic reprogramming in this functional reversal, emphasising that efferocytosis is essentially an immunometabolic intersection process precisely regulated by metabolism. By releasing various metabolites such as ATP, lactate, adenosine and sphingosine‐1‐phosphate (S1P), apoptotic tumour cells not only recruit tumour‐associated macrophages (TAMs) but also metabolically pre‐program their functions, inducing polarisation towards a pro‐tumourigenic M2‐like phenotype. During the recognition stage, tumour cells exploit metabolic abnormalities, such as glycosylation and lipid oxidation, to modify surface ‘eat‐me/don't‐eat‐me’ signals, thereby hijacking macrophage recognition and engulfment programs. Upon completion of engulfment, systemic reprogramming of amino acid, lipid and glucose metabolism occurs within macrophages. These metabolic alterations synergistically lock their immunosuppressive phenotype and establish a metabolic symbiosis between the tumour and stromal cells.

**Conclusions:**

Based on these mechanisms, this review further explores translational strategies targeting the efferocytic–metabolic axis, aiming to reprogram the immunosuppressive efferocytosis into immune‐activating events to overcome TME‐mediated immunosuppression and enhance current therapeutic efficacy. By deeply dissecting the metabolic regulatory networks of efferocytosis, we aim to pave new directions for cancer immunotherapy, achieving a paradigm shift from ‘metabolic hijacking’ to ‘metabolic interventional therapy’.

## Highlights

- Metabolic reprogramming allows tumour cells to hijack efferocytosis for immune evasion.
- Metabolite release recruits and reshapes macrophages into a pro‐tumourigenic phenotype.
- Abnormal glycosylation and lipid oxidation modulate ‘eat‐me/don't‐eat‐me’ signals to escape clearance.
- Post‐efferocytic metabolic shifts sustain M2‐like polarisation, making the efferocytic–metabolic axis a vital therapeutic target.

## Graphical abstract

This review systematically deconstructs the pathological efferocytic—metabolic axis in tumours, framing it as a three‐stage, self‐reinforcing cycle: from metabolite‐driven macrophage recruitment, through hijacked surface recognition, to post‐phagocytic metabolic reprogramming that locks in immunosuppression. We integrate definitive genetic evidence, delineate stage‐specific therapeutic strategies and propose that targeting this integrated metabolic network is key to reversing immune tolerance and restoring anti‐tumour immunity.

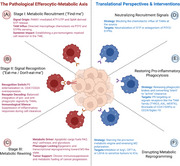

## 1 INTRODUCTION

Malignant tumours have emerged as a paramount global health concern and represent the leading cause of mortality among individuals under 70 years of age. According to data from the International Agency for Research on Cancer (IARC) in 2022, nearly 20 million new cancer cases and 9.7 million deaths occurred worldwide, and the incidence is projected to rise to 35 million by 2050.^1^ Despite transformative advances in diagnosis and therapy, current treatment modalities remain inadequate. Surgical resection is often unfeasible for patients with advanced disease or elderly individuals, chemotherapy frequently induces resistance and systemic toxicity,^2^ radiotherapy damages adjacent normal tissues,^3^ and immune checkpoint inhibitors (ICIs), though widely used, benefit only a subset of patients.^4^ Therefore, developing novel therapeutic strategies is an urgent priority.

Under physiological conditions, efferocytosis serves as a fundamental program for maintaining tissue homeostasis.^5^ This process involves the immunologically silent recognition, engulfment and degradation of apoptotic self‐cells by professional phagocytes, most notably macrophages. Typically coupled with the secretion of anti‐inflammatory cytokines, this process is indispensable for the resolution of inflammation and the promotion of tissue repair.^5^ In the tumour microenvironment (TME), however, the primary targets of macrophage‐mediated clearance shift towards apoptotic or dying tumour cells.^6^ Although this process employs similar molecular recognition modules, this aberrant clearance leads to a profound functional inversion: instead of facilitating wound healing, it drives immunosuppression, angiogenesis, metastasis and therapeutic resistance.^6^ This paradox raises a pivotal scientific question: Why do analogous clearance programs yield diametrically opposed pathological outcomes in the context of cancer? What are the deterministic factors governing this functional shift? Mounting evidence suggests that metabolic reprogramming acts as the central hub driving this functional reversal. The TME profoundly remodels the metabolic networks throughout the entire efferocytosis process,^7^ transforming apoptotic tumour cells from passive debris into active ‘metabolic signalling and resource hubs’ that release instructive cues and nutritional substrates. Specific metabolites released by these cells, such as ATP, lactate and adenosine, not only recruit macrophages,^8^ but also directly participate in orchestrating the immunosuppressive landscape.^9^ Ultimately, the immunological fate, a tolerance versus activation, may hinge upon the ‘quality’ and ‘quantity’ of the metabolite profile released by apoptotic cells, the metabolic fitness and polarisation of the recipient macrophage subsets, and the extent to which the equilibrium of surface recognition signals is hijacked.^8^, ^9^

Although the dual role of efferocytosis in cancer has gained significant attention, the underlying deterministic mechanisms remain to be fully elucidated. Current evidence suggests that the central hub driving the transition from physiological repair to pathological pro‐tumourigenesis is the systemic hijacking and remodelling of metabolic networks by the TME.^6^, ^7^

The TME does not passively accept the efferocytic process but actively subverts it by reshaping the metabolic dialogue between apoptotic cells and phagocytes.^7^ During the initiation phase, specific metabolite combinations released by apoptotic tumour cells, such as ATP, lactate and adenosine, serve as more than just ‘find‐me’ signals for recruitment.^10^, ^11^ They act as ‘metabolic instructions’ that pre‐program the phenotypic and functional direction of phagocytes.^9^, ^12^ The final immunological outcome, whether tolerance or activation, depends on the integration and balance of specific signalling pathways.^13^ This involves a dynamic interplay between ‘eat‐me’ and ‘don't‐eat‐me’ signals on the apoptotic‐cell surface, where their relative intensity and combination patterns are critical.^14^, ^15^ Simultaneously, the metabolic state of recipient phagocyte subsets, such as M2‐like macrophages or certain dendritic cells, profoundly influences how these apoptotic signals are interpreted.^16^, ^17^ Once engulfment is complete, the ingested material reshapes the phagocyte's metabolic network, activating intracellular pathways and inducing lasting epigenetic modifications that solidify a pro‐tumour phenotype.^18^, ^19^

Notably, this hijacked clearance program extends beyond professional phagocytes. In the TME, various non‐professional phagocytes, including tumour cells themselves and cancer‐associated fibroblasts (CAFs), are extensively involved in clearing apoptotic or dying cells.^20^ These activities are precisely regulated. For instance, macrophages can modulate the target selection of neighbouring epithelial cells by releasing soluble signals such as IGF‐1, thereby fine‐tuning local immune responses.^21^ When fibroblasts lead the clearance, the process relies on specific receptors and cytoskeletal remodelling, often driving their transformation into pro‐tumour states.^22^ Most critically, tumour cells can take the initiative through a mechanism termed emperitosis, where they actively internalise and eliminate cytotoxic immune cells to achieve direct immune evasion.^23^ Regardless of the specific mechanism, these trans‐boundary engulfment events lead to a common consequence: the profound metabolic and secretory reprogramming of the phagocyte, which reinforces the immunosuppressive landscape and enhances therapeutic resistance.

To resolve this complex network, this review adopts the perspective of ‘metabolic reprogramming’ to elucidate how tumours actively reshape the metabolic axis from death signal release to post‐engulfment digestion.^6^, ^24^, ^25^ We conceptualise this axis into three continuous and mutually reinforcing stages.

### 1.1 Phase I: Apoptotic tumour cells as metabolic messengers

Cell death in the TME is an active signalling event. Apoptotic tumour cells initiate metabolic programs to transform themselves into potent sources of diverse metabolites. These molecules form a ‘chemical language’ that goes beyond simple chemoattraction. While classic ‘find‐me’ signals like ATP and lysophosphatidylcholine (LPC) establish gradients to recruit phagocytes,^10^, ^11^ the TME is also enriched with ‘educator’ metabolites like lactate, adenosine, PGE2 and polyamines. For example, lactate not only serves as an energy substrate for tumour‐associated macrophages (TAMs) but also induces histone lactylation to directly reprogram the macrophage transcriptome towards immunosuppression.^12^, ^26^ Similarly, adenosine inhibits anti‐tumour pathways like interferon production via A2AR signalling^9^, ^27^ Thus, before physical contact occurs, macrophages are already pre‐programmed by these metabolites, setting an irreversible foundation for their subsequent pro‐tumour polarisation.

### 1.2 Phase II: Surface recognition and signal decoding via metabolic tags

Phagocytosis begins with the interpretation of the balance between ‘eat‐me’ and ‘don't‐eat‐me’ signals. In the TME, tumour cells utilise metabolic abnormalities to actively modify these surface tags. Examples include phosphatidylserine (PS) externalisation driven by lipid metabolic stress^13^ and abnormal glycosylation of CD24 or ICAM‐3 reflecting dysregulated glucose metabolism.^28^, ^29^ Furthermore, tumours employ a dual blockade strategy by overexpressing CD47 while using proteins like STC1 to suppress the display of calreticulin (CRT).^14^ Even when recognition occurs, downstream signalling through receptors like Stabilin‐1 or the TAM family is often distorted to activate STAT3 or NF‐κB.^16^, ^30^ Consequently, the recognition interface evolves from a simple ‘phagocytic switch’ into a complex ‘programming switch’ that domesticates macrophages with every contact.

### 1.3 Phase III: Post‐phagocytic systemic metabolic reprogramming

The completion of engulfment marks the beginning of a deeper functional hijacking. The ingested apoptotic cell acts as a ‘nutrient pack’ whose degradation products—amino acids, lipids and nucleic acids—flood the macrophage.^18^ In the amino acid axis, the conversion of arginine to putrescine via the Arg1–ODC axis fuels sustained phagocytosis and M2‐like polarisation.^19^ Simultaneously, tryptophan metabolism via IDO1 into kynurenine activates the aryl hydrocarbon receptor (AhR) to strengthen immunosuppression.^31^ Methionine provides a lasting impact by enabling DNMT3A‐mediated DNA methylation, which ‘locks’ pro‐tumour transcriptional programs into metabolic memory.^32^

Lipid metabolic reprogramming is equally vital, as the phagocytic load shifts macrophage metabolism towards fatty acid oxidation (FAO), activating the PPARγ–STAT6 axis to drive M2 gene transcription.^33^ Moreover, accumulated lipids can be reverse‐exported via ABCA1 to ‘feed’ proliferating tumour cells, establishing a metabolic symbiosis.^34^ Finally, the glycolysis–lactate axis connects energy metabolism to epigenetic control. Efferocytosis‐induced lactate drives the lactylation of promoters for genes like Arg1, solidifying transient metabolic states into long‐term pro‐tumour functions.^35^ In summary, post‐phagocytic metabolic reprogramming is a multi‐dimensional and hierarchical hijacking process. By integrating profound alterations in amino acid, lipid and glucose metabolism, this process not only satisfies the energetic and biosynthetic requirements of macrophages but also systematically rewrites their functional programs across multiple levels, including signal transduction, gene transcription and epigenetic memory. Consequently, these phagocytes are transformed into stable, active and self‐reinforcing pro‐tumour components within the tumour ecosystem.

A deeper understanding of this hijacked metabolic axis is driving the development of innovative therapeutic strategies that span the entire process of cell clearance. At the initiation stage, researchers are exploring the activation of the ATP–P2 × 7 signalling axis on macrophages to trigger pro‐inflammatory immune responses.^36^ Simultaneously, nanozymes capable of precisely catalysing the degradation of intratumoural lactate are being developed to reshape the TME from its metabolic source.^37^

Moving to the recognition stage, current clinical efforts are focused on blocking ‘don't‐eat‐me’ signals such as CD47 or CD24 using monoclonal antibodies,^15^, ^38^ as well as utilising pharmacological agents to induce the exposure of ‘eat‐me’ signals like CRT on the surface of tumour cells.^39^ More advanced therapeutic paradigms seek to directly reprogram macrophage functions through engineered phagocytic receptors or bispecific antibodies, thereby endowing them with entirely new recognition and killing logic.^40^

In the post‐phagocytic phase, intervention strategies focus on reversing the metabolic reprogramming of macrophages. This includes inhibiting arginase activity,^19^ blocking IDO1‐mediated tryptophan metabolism,^31^ or interfering with polyamine biosynthesis^41^ to alleviate immunosuppression. Furthermore, modulating lipid metabolism via PPARγ antagonists^42^ and disrupting the epigenetic locking of the M2 phenotype by lactylation have emerged as promising directions for reshaping macrophage function at the levels of energy and gene expression. Future breakthroughs will likely depend on the development of spatiotemporally precise combination therapies. For instance, combining these metabolic modulators with immune checkpoint blockade, chemotherapy or radiotherapy^7^ aims to multi‐dimensionally break the vicious cycle of ‘cell death, aberrant clearance, metabolic reprogramming and pro‐tumourigenesis’, ultimately reprogramming TAMs from accomplices of disease into active participants in anti‐tumour immunity.

In conclusion, the clearance of apoptotic tumour cells within the TME has evolved from a homeostatic program into a pathological engine driven by systemic metabolic reprogramming. This metabolic–efferocytic axis serves as a critical nexus linking cell death to immunometabolic dysregulation, revealing how tumours hijack host repair mechanisms for immune escape.

**FIGURE 1:**
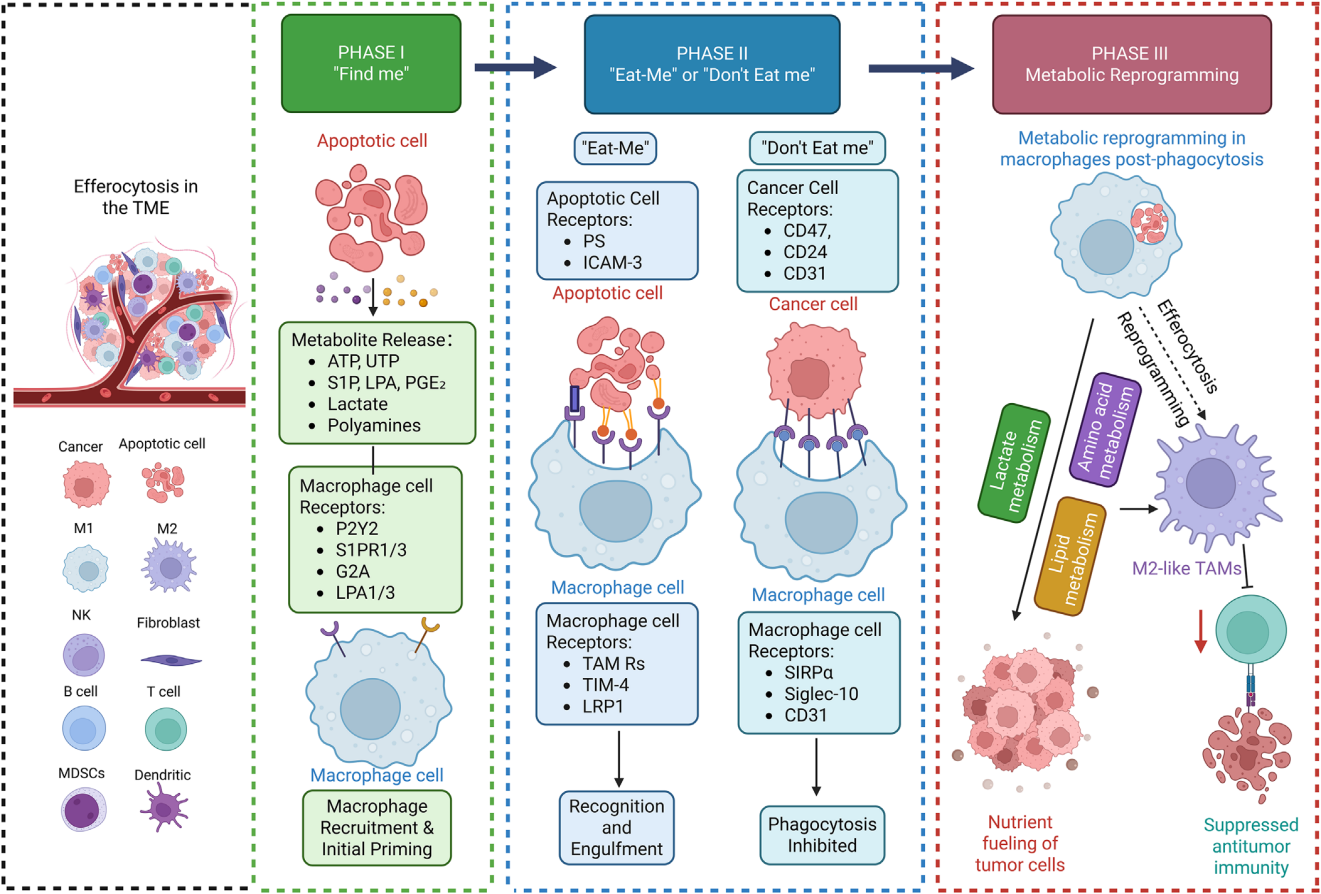
Systematic reprogramming of the efferocytic–metabolic axis within the tumour microenvironment. Phase I: ‘Find‐me’ and recruitment. Apoptotic cells within the TME initiate the process by releasing a diverse array of soluble ‘metabolic messengers’, including nucleotides (ATP, UTP) and various metabolites (S1P, LPA, PGE_2_, lactate and polyamines). These molecules establish chemotactic gradients recognised by specific macrophage receptors, such as P2Y2, S1PR1/3, G2A and LPA1/3, leading to active macrophage recruitment and initial functional priming at the apoptotic site. Phase II: Surface recognition and engulfment. This phase is governed by the molecular balance between pro‐phagocytic (‘eat‐me’) and anti‐phagocytic (‘don't‐eat‐me’) signals on the cell surface. ‘Eat‐me’ signalling: Macrophages recognise apoptotic cells through surface markers such as phosphatidylserine (PS) and ICAM‐3, sensed by receptors including TAM Rs (TYRO3, AXL, MERTK), TIM‐4 and LRP1 to trigger engulfment. ‘don't‐eat‐me’ signalling: Viable cancer cells evade clearance by overexpressing inhibitory checkpoints such as CD47, CD24 and CD31, which engage macrophage receptors (SIRPα, Siglec‐10 and CD31) to inhibit phagocytosis. Phase III: Metabolic reprogramming. Upon completion of engulfment, the internalised ‘apoptotic cargo’ triggers systemic internal metabolic rewiring within the macrophage. The integration of amino acid, lipid and lactate metabolism pathways drives polarisation towards a pro‐tumourigenic M2‐like phenotype. These M2‐like TAMs subsequently suppress anti‐tumour immunity and establish a symbiotic ‘nutrient fuelling’ loop that directly supports tumour progression. ICAM‐3, intercellular adhesion molecule 3; LPA, lysophosphatidic acid; LRP1, low‐density lipoprotein receptor‐related protein 1; PGE2, prostaglandin E2; PS, phosphatidylserine; Siglec‐10, sialic acid–binding Ig‐like lectin 10; SIRPα, signal regulatory protein alpha; S1P, sphingosine‐1‐phosphate; TAM, tumour‐associated macrophage; TME, tumour microenvironment; TIM‐4, T‐cell immunoglobulin and mucin‐domain containing‐4.

To resolve this complex network, this review adopts an integrative perspective to elucidate how tumours actively subvert the metabolic axis from initial signalling to post‐engulfment digestion. We conceptualise this process into three reinforcing stages that form the framework of this discussion: (1) the role of apoptotic cells as ‘metabolic messengers’ that recruit and pre‐program macrophages; (2) the decoding of surface ‘metabolic‐immune tags’ that hijack recognition logic; and (3) the post‐phagocytic metabolic and epigenetic rewiring that solidifies pro‐tumour phenotypes. By clarifying these mechanisms, we aim to provide a theoretical foundation for targeting this hijacked axis, ultimately transforming immunosuppressive clearance into a potent trigger for anti‐tumour immunity (Figure 1).

## 2 STAGE I: METABOLIC CUES GOVERNING APOPTOTIC‐CELL CLEARANCE: FROM RECRUITMENT TO FUNCTIONAL EDUCATION

A substantial spatial gap often exists between apoptotic cells and professional phagocytes, which delays prompt clearance of distant apoptotic corpses.^10^ To overcome this distance limitation, apoptotic cells undergo active metabolic reprogramming to release a series of structurally diverse and functionally precise metabolites. These molecules act as long‐range ‘metabolic messengers’ to construct a chemical instruction system that guides macrophage behaviour.^11^, ^43^, ^44^ These messengers, including nucleotides (ATP/UTP), sphingolipids (sphingosine‐1‐phosphate [S1P]), phospholipid derivatives (LPC, oxPLs), eicosanoids (PGE2), polyamines and lactate, collectively form a metabolic chemotactic gradient that precisely guides macrophages towards apoptotic sites.^10^, ^11^, ^44^, ^45^

Under physiological conditions, the core objective of this metabolic instruction system is to achieve ‘immunologically silent’ clearance. This involves efficient engulfment of apoptotic cells while activating macrophages in a pro‐resolving and tissue‐protective manner, thereby limiting inflammatory spread and bystander damage while initiating repair programs to maintain tissue homeostasis.^5^, ^46^ In the pathological context of cancer, however, this sophisticated metabolic communication network is systematically hijacked. By abnormally amplifying and remodelling these metabolic signals, tumour cells and their apoptotic debris not only enhance macrophage recruitment but also reprogram their metabolic and functional states. This subverts the clearance program into a pathological process driving immunosuppression, angiogenesis and therapeutic resistance.^47^, ^48^

Consequently, based on their core roles in the TME, these metabolic signals can be conceptualised into two continuous and often overlapping stages. First are the chemotactic ‘find‐me’ signals, such as ATP, LPC and S1P, which primarily mediate the spatial recruitment of macrophages.^10^, ^11^, ^44^ Second are the immunomodulatory metabolites, such as lactate, adenosine, PGE2 and polyamines, which are typically produced in large quantities by apoptotic or stressed tumour cells. Their core function transcends simple chemoattraction to ‘educate’ or reprogram macrophages and other immune cells through the induction of metabolic adaptations and epigenetic changes. This confers and solidifies a pro‐tumour phenotype, laying the foundation for a tolerant and immunosuppressive TME.^9^, ^48^, ^49^ Together, these two stages constitute a complete metabolic instruction chain through which apoptotic cells regulate the immune microenvironment.

### 2.1 Apoptotic‐derived metabolic signals: Instructions regulating macrophage efferocytosis

Apoptotic cells are active signalling sources in the TME, and their released metabolites constitute the core ‘metabolic language’ regulating the efferocytic process. These signals execute a systematic dual instruction: serving as chemotactic signals for spatial recruitment and as metabolic reprogramming signals that directly reshape the energy metabolism, epigenetic state and functional phenotype of phagocytes. The coupling of cell clearance with metabolic remodelling is a central hub connecting apoptosis to tumour immunosuppression.^50^

#### 2.1.1 Nucleotides: Metabolic switches for efferocytosis initiation and inflammatory reprogramming

In the early stages of cell death, apoptotic cells actively release nucleotides (ATP and UTP) through caspase‐mediated metabolic pathways, building a metabolic bridge between cell death and immune cell recruitment.^11^ The core of this process is the cleavage and activation of the plasma membrane channel Pannexin 1 (PANX1) by the metabolic effectors caspase‐3/7.^11^, ^44^ The cleaved PANX1 channels open in a quantised manner, leading to the massive release of intracellular ATP and UTP into the extracellular space to form local chemical gradients.^44^, ^45^

Released nucleotides (ATP/UTP) act as critical ‘find‐me’ signals by activating purinergic receptors on neighbouring macrophages to initiate directional chemotaxis. Research indicates that ATP and UTP primarily mediate chemotactic responses via the P2Y2 receptor to guide macrophages towards apoptotic sites. Depleting nucleotides or knocking out the P2Y2 receptor significantly impairs recruitment efficiency, establishing P2Y2 as the core receptor for ‘find‐me’ chemotaxis.^45^ Beyond chemotaxis, purinergic signalling directly promotes the adhesion and engulfment capabilities of phagocytes. For instance, various P2X and P2Y receptor agonists can upregulate the integrin Mac‐1 (CD11b/CD18) on macrophages, thereby enhancing their adhesion to apoptotic cells.^47^ In specific tissue environments, purinergic signals exhibit even more direct functions, such as in the brain where the P2Y6 receptor on TAMs can sense UDP released by apoptotic cells to directly initiate the phagocytic program.^51^

In the TME, the concentration of extracellular ATP (eATP) released by apoptotic tumour cells carries critical metabolic significance. Low concentrations of eATP primarily act as recruitment signals.^45^ However, when eATP accumulates to high local concentrations, it can be sensed by the P2 × 7 receptor on tumour‐infiltrating myeloid cells, including macrophages.^52^ Activation of P2 × 7R triggers profound immunometabolic reprogramming, such as assembly of the NLRP3 inflammasome and the release of pro‐inflammatory cytokines like IL‐1β.^36^, ^52^ This demonstrates that apoptotic nucleotides are not only recruitment signals but can also directly initiate inflammatory metabolic programs. Additionally, other cells in the TME, such as pyroptotic adipocytes, also release ATP to further amplify immunomodulatory effects.^53^ Notably, eATP in the TME can be rapidly hydrolysed by extracellular enzymes like CD39 into AMP and subsequently converted into adenosine.^36^ Adenosine is a potent immunosuppressive molecule that transmits strong anti‐inflammatory signals via the A2A receptor in macrophages, thereby inhibiting anti‐tumour immunity.^9^, ^27^ This metabolic conversion highlights the dynamic capacity of the TME to remodel metabolic signals.

#### 2.1.2 Sphingosine‐1‐phosphate: The lipid axis linking efferocytosis to metabolic polarisation

Apoptotic cells transform cell death into an active regulatory program by upregulating sphingosine kinase 1 and 2 (SphK1/2) and releasing the find‐me signal S1P.^54^, ^55^ This process transforms apoptosis from a simple clearance event into an active program that shapes the metabolic and immune phenotype of the TME.

The release of S1P creates a local chemical gradient that directly recruits macrophages for efferocytosis.^54^ In the TME, this population undergoes profound metabolic reprogramming driven by S1P signalling.^55^, ^56^ S1PR signalling shifts the macrophage phenotype from pro‐inflammatory M1 towards pro‐repair M2^55^ and activates the expression of angiogenesis‐related genes via HIF‐1α.^57^

This metabolic shift produces multiple pro‐tumour effects. M2‐polarised macrophages secrete angiogenic factors and release immunosuppressive factors that inhibit the activity of CD8+ T cells.^58^, ^59^ Polarisation driven by S1P is a key pathway for this functional reprogramming, which can be reversed by the knockdown of SphK2 in tumour cells.^55^ Furthermore, continuous efferocytosis exposes macrophages to chronic S1P signalling, establishing a self‐reinforcing loop of immunosuppressive and angiogenic states. Notably, S1P signalling can also activate erythropoietin (EPO) signalling, which activates PPARγ to further enhance efferocytic capacity and immune tolerance.^49^ In cancers such as hepatocellular carcinoma, the NEK2‐driven S1P synthesis pathway directly confers tumour resistance to ICIs.^59^ Targeting this axis, including the inhibition of SphK1 activity or blocking S1P receptors, represents a potential strategy to reverse immunosuppression.^60^, ^61^

#### 2.1.3 LPC/LPA: Lipid metabolic signals regulating specific macrophage phenotypes

LPC and its metabolite lysophosphatidic acid (LPA) constitute a complete signal axis from death signal transmission to immunometabolic remodelling. Upon apoptosis, caspase‐3 activates iPLA_2_ to catalyse phospholipid hydrolysis, generating the find‐me signal LPC.^10^ LPC is actively transported extracellularly via ABCA1, whose function directly determines the efficiency of LPC signalling.^50^ In the microenvironment, released LPC can be further metabolised by autotaxin (ATX) into the more bioactive LPA. Additionally, secretory phospholipase A2 (sPLA_2_) in membrane microvesicles can directly hydrolyse phospholipids to generate LPA.^48^

Physiologically, LPA drives directional migration by activating the LPA1 receptor on macrophages.^62^ In the TME, this program is hijacked into a pro‐tumour metabolic engine. In glioblastoma, tumour cells induce TAMs (including both resident microglia and recruited macrophages) to overexpress ATX, and the resulting LPA acts on the tumour cells' own LPA1 receptors to enhance proliferation and migration.^63^

Crucially, different LPA species induce distinct metabolic outcomes. 20:4 LPA (arachidonic acid‐LPA) activates RHO/RAC1 and p38 MAPK to drive migration, while 18:0 LPA (stearic acid‐LPA) activates AKT survival signalling.^64^ In colorectal cancer, the enzyme Agpat4 regulates LPA metabolism to activate p38/p65 NF‐κB signalling in macrophages, driving their polarisation towards the immunosuppressive M2‐like phenotype and inhibiting T cell activity.^65^

Interestingly, inhibiting Agpat4 in tumour cells leads to the release of LPA that instead polarises macrophages towards the M1 phenotype via LPA_1_/LPA_3_ receptors.^66^ Notably, basic metabolic dysregulation, such as liver AKAP1/PKA axis defects, can worsen the pre‐tumour environment by promoting LPA generation.^66^

#### 2.1.4 Oxidised phospholipids: Damage signals driving pro‐inflammatory metabolic reprogramming

Oxidised phospholipids (oxPLs) act as active signalling hubs that systematically reprogram macrophage lipid metabolism and function.^43^, ^67^ First, oxPLs act as ‘damage signals’ to initiate macrophage recruitment and recognition.^68^ Apoptotic cells generate oxPLs through oxidative modification of membrane lipids, which act as damage‐associated molecular patterns (DAMPs) to recruit macrophages to the site of death.^67^ Recruited macrophages recognise and bind oxPLs via TLR4 and the scavenger receptor CD36.^67^, ^68^

CD36‐mediated endocytosis of oxPLs is critical for driving macrophage metabolic reprogramming. Ingested oxPLs accumulate within the macrophage, altering its lipidomic profile and accumulating pro‐inflammatory lipid mediators.^68^, ^69^ Lipidomic research shows that different oxPL species uniquely reshape the lipid profile and can synergise with LPS.^69^ In the TME, macrophages ingesting oxPLs from ferroptotic cells can activate the NLRP3 inflammasome, resulting in massive IL‐1β secretion and facilitating tumour invasion and metastasis.^70^ In lung cancer, TAMs support tumour fate by providing L‐carnitine to support FAO in cancer stem cells via the CPT1A axis, enhancing their antioxidant capacity and therapy resistance.^71^ This highlights abnormal lipid metabolism as a critical regulator of immunotherapy efficacy.^72^ Targeting oxPLs and their regulatory pathways has become a potential strategy for intervention.^43^, ^70^, ^71^

### 2.2 Microenvironmental immunomodulatory metabolites: Programming post‐efferocytic homeostasis

The metabolites in this section program the metabolic state of the immune microenvironment after clearance, ensuring that macrophage functional output favours tolerant clearance and pro‐tumour homeostasis.

#### 2.2.1 Adenosine: An immunosuppressive messenger converted from efferocytic ATP

Following engulfment, macrophages undergo endogenous metabolic remodelling. To meet energetic demands, they upregulate solute carriers and initiate a unique program of aerobic glycolysis driven by SLC2A1‐mediated glucose uptake.^17^ Meanwhile, the glycolytic byproduct lactate is released via SLC16A1 to contribute to a local anti‐inflammatory state.^17^ Concurrently, fatty acids from apoptotic cells are utilised by macrophage mitochondria via β‐oxidation, which enhances NAD+ levels and activates epigenetic regulators like SIRTUIN1 to promote the anti‐inflammatory cytokine IL‐10.^46^

The second pathway involves the active hijacking and conversion of extracellular signals by the TME. eATP released by apoptotic cells is rapidly hydrolysed by the ectonucleotidases CD39/CD73 into adenosine.^9^ Adenosine is a potent immunosuppressive metabolite that promotes macrophage polarisation towards the M2‐like phenotype and suppresses effector T cell function through A2A receptor signalling.^9^ This pathway is central to resistance in many tumours. For example, in hepatocellular carcinoma, sorafenib‐induced mitochondrial damage releases ATP that synergises with mitochondrial DNA to drive M2 polarisation via TLR9.^73^ In ovarian cancer metastasis, adipocyte pyroptosis releases ATP that establishes a pro‐TME after being metabolised into adenosine.^53^ Targeting this node, such as by inhibiting CD39 or activating the P2 × 7‐inflammasome–IL‐18 axis, has become a strategy for reversing immunosuppression.^36^ Similarly, inducing immunogenic cell death (ICD) to release ATP can polarise macrophages to the anti‐tumour M1 phenotype.^74^, ^75^

#### 2.2.2 Lactate: Driving pro‐tumour polarisation via lactylation

Lactate in the TME transcends its role as a waste product to educate efferocytic macrophages.^12^, ^26^ Tumour cells generate lactate via the Warburg effect,^12^ while macrophages performing efferocytosis also significantly enhance glycolysis to generate their own lactate via SLC transporters.^17^

High lactate levels drive protein lactylation, directly rewriting macrophage epigenetic programs. In tumour‐infiltrating myeloid cells, lactate induces H3K18la, which upregulates the methyltransferase METTL3. METTL3 then enhances the translation of JAK1 mRNA via m6A modification, continuously activating the JAK1/STAT3 pathway to solidify immunosuppressive functions.^76^ In pancreatic cancer, lactate induces ENSA‐K63la, activating the STAT3/CCL2 axis to drive TAM transcriptional reprogramming.^35^ Furthermore, lactate can target and inhibit MAVS proteins, blocking type I interferon production.^77^ Educated macrophages then turn into tumour accomplices, secreting factors like CCL2 and IL‐6.^78^, ^79^ Tumour cells maintain this environment through mechanisms such as SETDB1‐mediated stability of MCT1.^80^ Targeting this axis via LDHA inhibition,^81^, ^82^ blocking MCT1/4,^83^ or using nano‐platforms^37^ is a key strategy for reversing the pro‐tumour phenotype.

#### 2.2.3 Prostaglandin E2: A lipid mediator of post‐efferocytic metabolic suppression

PGE2 serves as a powerful metabolic educator, reprograming efferocytic macrophages through EP2 and EP4 receptors.^84^ Apoptotic cells upregulate COX‐2 and activate phospholipase A2 to generate PGE2.^84^ PGE2 signalling drives M2‐like polarisation and the secretion of anti‐inflammatory factors like interleukin‐10 (IL‐10) and transforming growth factor beta (TGF‐β).^85^

In hepatocellular carcinoma, PGE2 transforms TAMs into a pro‐angiogenic CX3CR1+ subpopulation,^86^ while in pancreatic cancer, it synergises with tumour necrosis factor (TNF) to induce IL‐1β.^87^ Beyond phenotype, PGE2 impairs the bioenergetic metabolism and ribosome biosynthesis of infiltrating immune cells, fundamentally weakening their function.^88^ Targeting this axis has been shown to reduce MDSCs^89^ and promote M1 repolarisation^89^, ^90^ while enhancing T cell infiltration.^90^, ^91^ This can synergise with chemotherapy,^92^ photothermal therapy^93^ and immune checkpoint blockade.

#### 2.2.4 Polyamines: Engines for sustained efferocytosis and epigenetic reprogramming

Polyamines are critical regulators of the immune microenvironment.^94^ Physiologically, macrophages convert apoptotic‐derived arginine into putrescine to fuel ‘continual efferocytosis’ through Rac1 activation.^19^ In the TME, cancer cells hijack this cycle. In breast cancer, TAMs take up tumour‐derived arginine and convert it into putrescine via Arg1/ODC.^41^, ^95^ Accumulated putrescine solidifies M2‐like polarisation through p53‐dependent DNA demethylation.^41^

In hepatocellular and pancreatic cancers, spermidine directly induces M2 polarisation and impairs CD8+ T cell function through the PI3K‐Akt‐mTOR‐S6K pathway.^96^, ^97^ Additionally, tumour‐derived N1‐acetylated polyamines can enhance mitochondrial respiration in myeloid cells and induce CCL1+ macrophages that recruit Tregs.^98^, ^99^ Targeting this axis, via arginine–polyamine axis disruption,^41^ inhibiting spermidine synthesis,^98^ or blocking acetylated polyamine transport,^98^ effectively reprograms the TME.

### 2.3 Summary: Targeting metabolic regulatory nodes in the initial stage of phagocytosis

**FIGURE 2:**
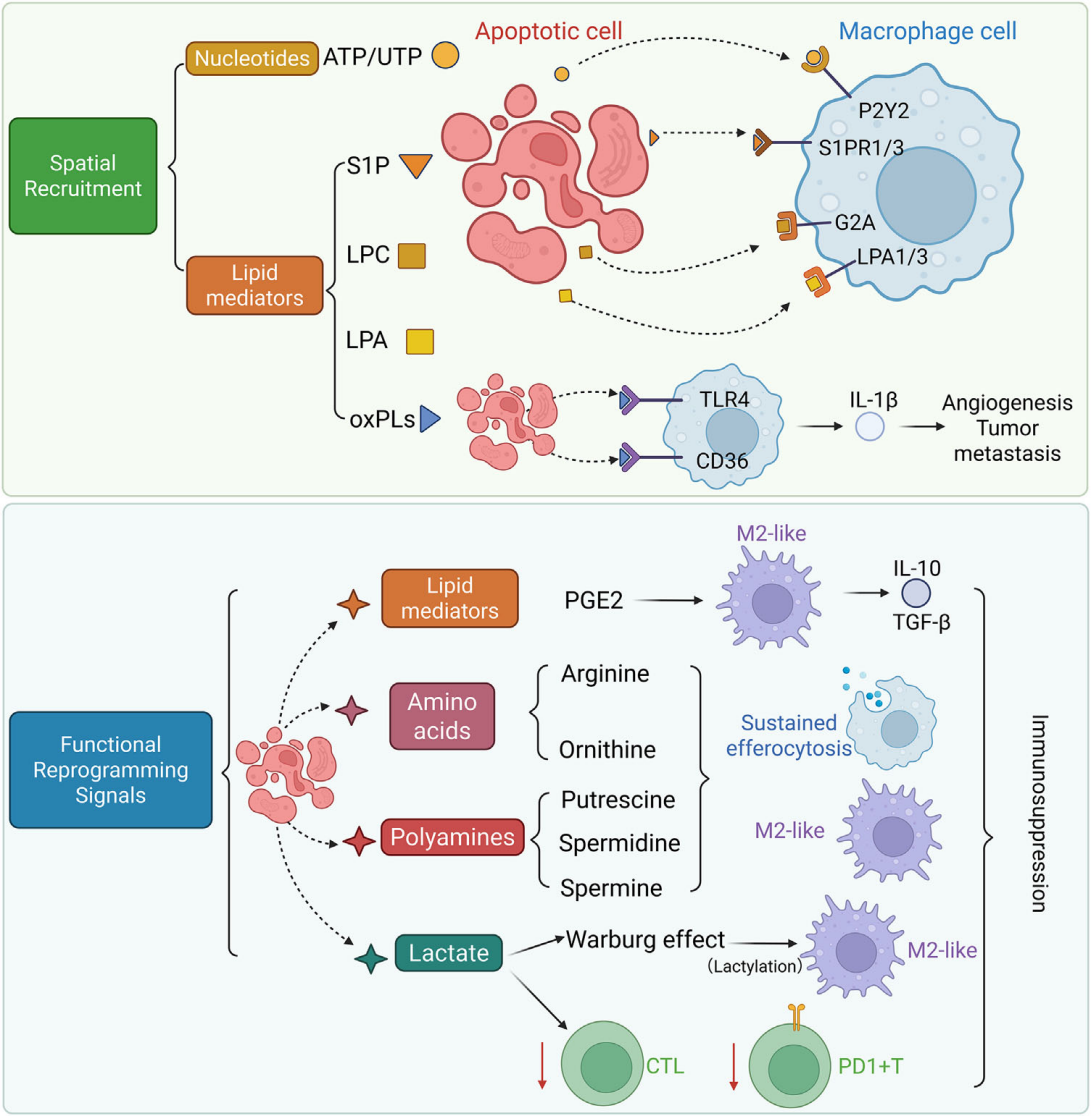
Spatial recruitment and functional reprogramming signals driving immunosuppressive efferocytosis in the tumour microenvironment (TME). Upper panel: Apoptotic tumour cells release canonical ‘find‐me’ signals, categorised into nucleotides (ATP/UTP) and lipid mediators (sphingosine‐1‐phosphate [S1P], lysophosphatidylcholine [LPC], lysophosphatidic acid [LPA]). These signals are recognised by specific macrophage receptors—P2Y2, S1PR1/3, G2A and LPA1/3—to facilitate the spatial recruitment of macrophages. Simultaneously, oxidised phospholipids (oxPLs) engage TLR4 and CD36, triggering the secretion of IL‐1β and subsequently promoting angiogenesis and tumour metastasis. Lower panel: Beyond recruitment, apoptotic cells release functional reprogramming signals that modulate the TME. Lipid mediators like PGE2 induce M2‐like polarisation and the release of anti‐inflammatory cytokines (IL‐10, TGF‐β). Metabolites such as amino acids (arginine, ornithine) and polyamines (putrescine, spermidine, spermine) support sustained efferocytosis. Notably, lactate promotes the Warburg effect and macrophage lactylation, further driving M2‐like differentiation. These combined signals culminate in potent immunosuppression by inhibiting cytotoxic T lymphocytes (CTLs) and increasing PD‐1^+^ T cells, collectively fostering immune tolerance and tumour progression.

In the TME, metabolites actively released by apoptotic cells constitute a sophisticated chemical instruction system that regulates macrophage efferocytosis.^11^, ^12^, ^17^, ^43^, ^44^, ^49^ These instructions perform dual functions: acting as chemotactic find‐me signals (e.g., ATP, LPC, S1P) to recruit macrophages,^10^, ^11^, ^44^ and serving as metabolic reprogramming signals during efferocytosis.^17^, ^19^, ^35^, ^41^, ^76^ This process ensures that macrophages maintain a pro‐tumour phenotype following efferocytosis.^85^, ^87^, ^96^, ^98^ Targeting these critical nodes provides a promising paradigm for the metabolic intervention of the tumour immune microenvironment (Figure 2).^36^, ^37^, ^60^, ^90^, ^98^

## 3 PHASE II: RECOGNITION OF APOPTOTIC CELLS VIA ‘EAT‐ME’ AND ‘DON'T EAT‐ME’ SIGNALS

Apoptotic tumour cells release a surge of ‘find‐me’ signals to attract TAMs. The critical step of phagocytosis lies in the precise recognition between ‘eat‐me’ signals displayed on the apoptotic‐cell surface and their corresponding receptors on macrophages.

### 3.1 ‘Eat‐me’ signals and their receptor networks

#### 3.1.1 Classic ‘eat‐me’ signals: recognition and regulation of phosphatidylserine

##### Mechanisms of PS exposure and functional hijacking in tumour

Immunosuppression in healthy cells: PS is strictly confined to the inner leaflet of the plasma membrane. This asymmetric distribution is maintained by the continuous activity of the ATP‐dependent flippase ATP11C/CDC50A complex, a highly ordered and energy‐consuming metabolic process.^100^ From a metabolic perspective, the initiation of apoptosis triggers a profound remodelling of membrane lipid metabolism via the caspase cascade. On one hand, caspase‐3 mediates the inactivation of ATP11C, blocking the ATP‐consuming inwards flipping of phospholipids. On the other hand, the scramblase Xkr8, activated through post‐translational modifications such as phosphorylation, initiates energy‐independent bidirectional random flipping.^101^ The synergy between the closing of the ‘energy gate’ and the opening of the ‘diffusion channel’ constitutes the active and controlled metabolic event of PS externalisation, which fundamentally alters the metabolic flux balance required to maintain membrane asymmetry.

Externalised PS serves as a pivotal metabolic‐immune signalling molecule whose recognition is the rate‐limiting step of efferocytosis.^102^ In the TME, persistent apoptosis and PS exposure systematically reprogram the metabolic and functional states of macrophages, ‘taming’ them towards a pro‐tumour phenotype:

1. PS directly activates the downstream FAK–SRC–STAT3 signalling axis by binding to specific receptors (e.g., TIM‐4, PSR) on macrophages. This pathway not only transmits proliferation and polarisation signals but also upregulates the histone demethylase JMJD3. Through epigenetic remodelling, this solidifies the gene expression profile of M2‐like macrophages, forming a lasting immunosuppressive metabolic memory.^103^
2. PS acts as a ‘metabolic tag’ recognised by the soluble bridging protein Gas6. Once bound, Gas6 functions as a metabolic signal amplifier, delivering signals with high affinity to receptor tyrosine kinases such as Mer and Axl, thereby integrating extracellular cues with intracellular pro‐survival and anti‐inflammatory metabolic pathways.^102^

Consequently, tumour cells hijack the PS exposure process—originally intended for homeostasis—into a chronic metabolic instruction that drives immunosuppression.^13^ Sustained PS signalling not only guides macrophages to clear apoptotic cells silently but also systematically promotes the polarisation, infiltration and activation of TAMs towards an M2‐like phenotype, maintaining an immunosuppressive environment. Experimental evidence supports the core status of this metabolic–signalling axis: exogenous PS supply can accelerate tumour growth, while intervening in PS exposure or its downstream recognition can break this harmful cycle.^103^, ^104^ This highlights the potential of targeting ‘eat‐me’ signal recognition as a metabolic checkpoint for reshaping the tumour immune microenvironment.

##### Direct receptor mechanisms for PS recognition: hubs for metabolic sensing and signal integration

The recognition of PS by TAMs is a critical metabolic–signalling conversion node. This process involves more than simple ligand–receptor binding; macrophages utilise a receptor network to precisely sense and integrate signals of homeostatic imbalance from apoptotic cells, initiating specific functional and metabolic programs.^102^ These mechanisms are categorised into direct binding and indirect bridging, with direct receptors forming the primary line of specialised sensing.

###### Direct recognition receptors: First‐line sensors of homeostatic signals

Receptors such as TIM‐4, BAI1 and the Stabilin family act as ‘homeostasis sensors’ by binding directly to externalised PS. They not only initiate efferocytosis but also trigger downstream signals that profoundly reprogram the metabolic pathways and immune functions of macrophages, a process significantly distorted in the TME.^102^

1. TIM receptors (TIM‐4): Functional inversion from surveillance to suppression TIM‐4 directly recognises PS on macrophages. In early‐stage tumours, TIM‐4^+^ macrophages activate specific programs, including the upregulation of antigen‐processing genes and the delayed acidification of phagosomes, which facilitates antigen preservation and cross‐presentation to activate CD8^+^ T cells.^105^ However, in established tumours, this function is hijacked. Chronic PS exposure transforms TIM‐4‐mediated engulfment into a persistent stimulus that drives macrophages towards immunosuppression. Furthermore, TIM‐4 can directly recognise and inhibit activated cytotoxic T cells expressing PS, forming a ‘functional entrapment’ mechanism that directly weakens anti‐tumour immunity.^106^ Targeting TIM‐4 can relieve this inhibition and synergise with PD‐1 blockade to reshape anti‐tumour immunity.^107^
2. BAI1: Epigenetic silencing and functional inactivation BAI1 is a vital receptor for PS‐dependent phagocytosis, playing a core role in maintaining tissue homeostasis and clearing pathogens.^108^, ^109^ In tumours, its function is often suppressed. For example, in specific glioblastoma subtypes, the BAI1 gene undergoes epigenetic silencing via promoter methylation, which correlates with increased infiltration of TAMs and Tregs.^110^ Emerging engineering strategies, such as the chimeric efferocyte receptor (CHEF), fuse the PS‐recognition domain of BAI1 with intracellular signalling modules to enhance phagocytic capacity and modulate macrophage states, improving disease control in preclinical models.^40^
3. Stabilin receptors: Divergent functional outputs of homologous receptors while both are direct PS receptors, Stabilin‐1 and Stabilin‐2 guide macrophages towards vastly different functional outcomes in tumours.^111^, ^112^ Stabilin‐2‐mediated engulfment is typically coupled with anti‐inflammatory programs, such as inducing IL‐10 expression via the p38 MAPK–Pbx1 axis to promote inflammation resolution.^113^ Conversely, Stabilin‐1 is highly expressed on TAMs as a core immunosuppressive regulator. It promotes tumour progression by activating the IKK–NF‐κB pathway,^30^ driving M2 polarisation and inhibiting T cell function through its soluble form.^114^ Targeting Stabilin‐1 (e.g., using the bexmarilimab antibody) can reprogram macrophages from immunosuppressive to immunostimulatory states, enhancing antigen presentation and T cell activation.^115^, ^116^

In summary, direct PS receptors form a sophisticated ‘first‐line sensing and decision‐making network’. Under physiological conditions, they ensure the silent clearance of apoptotic cells.^13^ In the TME, this balance is broken: the surveillance‐potential receptor TIM‐4 is hijacked, the homeostatic receptor BAI1 is silenced and the tolerance‐promoting receptor Stabilin‐1 is overactivated. This imbalance is the molecular foundation by which tumours convert ‘eat‐me’ signals into tools for maintaining an immunosuppressive metabolic microenvironment.^117^, ^118^

##### TAM receptor tyrosine kinases: the indirect recognition axis and intervention

TAM receptors (Tyro3, Axl, MerTK) recognise PS indirectly through bridging ligands such as Gas6 and Protein S, acting as critical signal amplifiers.^119^ In the TME, TAM receptors are continuously activated, driving macrophages towards an M2‐like phenotype and promoting the secretion of immunosuppressive mediators.^16^ Intervention strategies targeting TAM receptors include small‐molecule kinase inhibitors,^120^ targeted protein degraders^121^ and ligand‐based CAR‐T cell therapies.^122^ TAM receptor activity is also regulated by metalloproteinase‐mediated shedding, which is associated with therapeutic resistance.^123^ Furthermore, strategies targeting specific TAM subsets^124^ or utilising Fc‐enhanced antibodies to clear Tregs via TAMs represent more refined approaches.^125^

From a metabolic perspective, these receptors constitute a signal‐decoding network that converts PS exposure into specific intracellular signalling and epigenetic reprogramming instructions. In tumours, the output of this network is tilted towards immunosuppression. Therefore, inhibiting PS signalling or degrading related receptors aims to break the tumour‐driven signalling cycle and transform phagocytosis into a process that supports effective anti‐tumour immunity.

#### 3.1.2 Calreticulin: An ER stress‐driven metabolic‐immune signal and its recognition

CRT is a key ‘eat‐me’ signal whose surface exposure constitutes a highly regulated metabolic‐immune checkpoint. Unlike PS, CRT translocation is not a universal marker of apoptosis but is closely linked to endoplasmic reticulum (ER) stress, the unfolded protein response and specific cell death programs like ICD.^126^ Macrophages recognise surface‐exposed CRT via the low‐density lipoprotein (LDL) receptor‐related protein LRP1/CD91, a decisive metabolic step that shapes the polarisation of subsequent anti‐tumour immune responses.^127^

##### Metabolic antagonism between CRT and CD47

Under homeostatic conditions, the pro‐phagocytic signal of CRT is balanced by the ‘don't‐eat‐me’ signal of CD47 to maintain self‐tolerance.^127^ Tumour cells break this balance through metabolic reprogramming, overexpressing CD47 to camouflage themselves. Many tumours exhibit basal CRT exposure due to persistent metabolic and ER stress.^128^ Resistance to phagocytosis in these cases arises not from a lack of CRT, but because high CD47 levels counteract CRT‐mediated triggers.^128^ Thus, CD47 blockade therapies function by de‐repressing CRT signals, allowing macrophages to ‘sense’ and respond to the tumour's abnormal metabolic state.

##### CRT exposure as an ‘output signal’ of metabolic stress

The translocation of CRT to the cell surface is an active response to metabolic interference. Therapeutic strategies such as specific chemotherapies, photodynamic/photothermal therapies and ferroptosis‐inducing protocols trigger ICD by disrupting metabolic homeostasis, leading to significant CRT exposure.^129^, ^130^ This induced CRT exposure synergises strongly with CD47 blockade to enhance phagocytosis.^39^ Interestingly, activated macrophages can also downregulate tumour CD47 and upregulate their own CRT to selectively clear tumour cells, suggesting bidirectional metabolic signalling.^131^

##### Tumour metabolic evasion: Interfering with CRT transport

Tumours have evolved metabolic strategies to weaken this ‘eat‐me’ signal. For instance, the inflammatory regulator A20 and the glycoprotein Stanniocalcin 1 (STC1) are upregulated in various cancers. They interfere with the transport of CRT from the ER to the cell surface; specifically, STC1 binds CRT and retains it intracellularly, inhibiting phagocytic recognition.^14^, ^132^ This highlights how tumours hijack protein secretion and transport pathways to achieve immune escape.

##### Targeting the CRT–CD47 axis via metabolic reprogramming

Emerging interventions aim to precisely regulate this axis. Strategies include using nanotechnology to deliver siRNA for CD47 silencing,^133^ or engineering extracellular vesicles and smart hydrogels to actively induce CRT exposure.^134^, ^135^ For example, an implantable hydrogel can remodel TME metabolic features to relieve hypoxia while upregulating tumour CRT and downregulating CD47, thereby systematically reprogramming macrophage function and stimulating adaptive immunity.^135^ These strategies aim to actively reprogram the immunogenic metabolic state of tumour cells and enhance macrophage recognition efficiency.

#### 3.1.3 Integrins: Adhesion receptors mediating phagocytic anchoring and immune regulation

Effective clearance of apoptotic cells depends on the stable anchoring provided by adhesion receptors such as integrins αvβ3 and αvβ5. In the TME, this physical anchoring mechanism is often hijacked by tumour cells.

##### Tumour hijacking of the integrin axis: From anchoring to signalling

Tumour cells remodel the local microenvironment by secreting extracellular matrix proteins like osteopontin or releasing extracellular vesicles carrying bridging molecules such as MFGE8.^136^, ^137^, ^138^ These ligands not only mediate macrophage recruitment and adhesion but also activate downstream pathways like STAT3, converting mechanical anchoring into biological instructions for immunosuppressive phagocytosis and M2 polarisation.^138^, ^139^ For example, osteopontin secreted by glioblastoma stem cells binds to TAMs αvβ3/αvβ5, simultaneously recruiting TAMs and transmitting signals to maintain their pro‐tumour phenotype.^136^, ^137^

Colorectal cancer cells deliver MFGE8 via exosomes to bridge PS on apoptotic cells with αvβ3 on macrophages.^138^ This ‘forced ligand–receptor coupling’ triggers the activation of the Src–FAK–STAT3 axis, reprogramming integrins from passive ‘adhesion switches’ into active ‘pro‐phagocytic signal amplifiers’. Consequently, efferocytosis—originally for homeostasis—is twisted into a pathological process that clears chemotherapy‐induced apoptotic cells and promotes therapeutic resistance.^138^

##### Therapeutic redirection of integrin signalling

Strategies focus on redirecting or reprogramming macrophage functions. One approach is to utilise the high expression of integrins on TAMs, using anti‐αvβ3 antibodies to ‘arm’ them as killing units that attack tumour cells via antibody‐dependent cellular cytotoxicity (ADCC).^140^, ^141^ Another is the use of specific peptides (e.g., 7aaRGD) or targeted delivery systems to block the SPP1/integrin axis, reversing the immunosuppressive phenotype of macrophages and synergising with ICIs.^142^, ^143^ Integrin signalling is context‐dependent; for instance, the protease legumain can negatively regulate the JAK1/STAT1 pathway through interaction with αvβ3, and its deficiency promotes a pro‐inflammatory state.^144^

#### 3.1.4 CD36: A multifunctional receptor linking lipid sensing to phagocytosis

As a class B scavenger receptor, CD36 plays a dual role in the TME: it is both a phagocytic receptor recognising ‘eat‐me’ signals and a sensor driving immunosuppressive metabolic reprogramming.^33^

##### The dual function of CD36

As a phagocytic receptor, CD36 directly recognises oxidised lipid ligands on apoptotic cells, which is the foundational mechanism for its mediation of efferocytosis.^145^ In the TME, this function is critical; for example, CD36 is required for the effective clearance of apoptotic tumour cells by metastasis‐associated macrophages.^146^

However, CD36‐mediated abnormal lipid uptake also drives metabolic reprogramming that suppresses immunostimulation.^33^, ^146^ TAMs utilise high CD36 expression to ingest tumour‐derived lipids, leading to intracellular accumulation. This enhances FAO, driving mitochondrial oxidative phosphorylation and ROS generation, which activates pathways like STAT6 and PPAR‐γ to systematically promote M2‐like polarisation.^33^, ^42^

This metabolic state directly impairs immune responses: CD36 signalling inhibits type I interferon production via the p38 MAPK pathway, weakening the ability of macrophages to support anti‐tumour T cell responses.^147^ In p53‐deficient hepatocellular carcinoma, cancer stem cells secrete IL‐34 to upregulate CD36 in TAMs, forcibly driving lipid metabolic remodelling and immune escape.^148^ The biological function of CD36 is highly context‐dependent; it can show protective interactions in fatty liver disease^149^ or promote tumour progression in papillary thyroid cancer via osteopontin secretion.^150^ Targeting CD36 aims to intervene in this abnormal metabolic reprogramming, reducing pathological lipid accumulation and reversing M2 polarisation.^151^

### 3.2 ‘Don't‐eat‐me’ signals and innate immune checkpoints

The precise recognition of apoptotic cells by macrophages acts as a switch for the immune response. This process is governed by the balance between activating ‘eat‐me’ signals and inhibitory ‘don't‐eat‐me’ signals.

#### 3.2.1 The CD47–SIRPα axis: The core inhibitory checkpoint

In the TME, cancer cells escape innate immune surveillance by overexpressing ‘don't‐eat‐me’ signals, tilting the balance of recognition. The CD47–SIRPα axis is the most well‐characterised innate immune checkpoint and is a cornerstone of tumour immune evasion.^38^

CD47 is a transmembrane protein that binds to SIRPα on macrophages to transmit a potent ‘don't‐eat‐me’ signal, a mechanism vital for self‐tolerance in healthy tissues.^38^, ^152^ During physiological apoptosis, cells downregulate CD47 to ensure safe clearance. Tumour cells, however, overexpress CD47 to mimic ‘normal self’ and actively inhibit macrophage phagocytosis.^152^, ^153^ In triple‐negative breast cancer, high CD47 expression is closely linked to the formation of an immunosuppressive microenvironment.^154^

Mechanistically, the CD47–SIRPα axis relies on the regulation of SHP2. Binding of CD47 to SIRPα triggers the de‐neddylation of SHP2, activating its phosphatase activity to dephosphorylate critical substrates required for phagocytic cup formation, thereby strongly inhibiting the initiation of engulfment.^155^ Therapeutic strategies aim to ‘reboot’ macrophage phagocytosis by blocking this axis. Various anti‐CD47 antibodies and SIRPα fusion proteins are currently in clinical trials for gastric cancer and haematological malignancies.^38^ Innovative approaches combine blockade with reprogramming: for example, using hybrid nanovesicles to deliver CD47‐blocking peptides (RS17) while reprogramming TAMs towards an M1‐like phenotype,^153^ or using antibody–oligonucleotide conjugates to combine anti‐CD47 antibodies with microRNA‐34a.^154^

#### 3.2.2 The CD31 (PECAM‐1) pathway: Dual signalling in recognition and metabolic adaptation

CD31 (PECAM‐1) is an important inhibitory signal molecule whose function transcends traditional adhesion. In the TME, it regulates both immune recognition and tumour metabolic adaptation.^156^, ^157^

Surface expression of CD31 transmits a strong anti‐phagocytic instruction. In angiosarcoma, CD31‐low subclusters exhibit weakened endothelial characteristics but enhanced tumourigenicity and resistance to doxorubicin.^156^ This phenomenon reveals a tight link between CD31 signalling and metabolic reprogramming: CD31 downregulation triggers the YAP signalling pathway, inducing antioxidant enzymes that allow CD31‐low cells to clear ROS and survive chemotherapy‐induced stress.^156^ CD31 also regulates intercellular communication; in hepatocellular carcinoma, CD31+ endothelial cells secrete IL‐4 to polarise macrophages towards a pro‐tumour M2 phenotype, a process correlated with poor prognosis.^158^, ^159^

Therapeutic strategies targeting CD31 have two dimensions. Directly blocking its ‘don't‐eat‐me’ function may restore phagocytosis, while intervening in its downstream metabolic adaptations can reverse resistance. For example, using pazopanib to inhibit YAP signalling can re‐sensitise CD31‐low cells to chemotherapy.^156^ In breast cancer, the compound AGS‐30 inhibits M2 polarisation and downregulates pro‐angiogenic molecules including CD31 to suppress tumour growth.^160^

#### 3.2.3 The CD24–Siglec‐10 pathway: Glycosylation‐driven recognition blockade

The CD24–Siglec‐10 axis is a critical innate immune checkpoint that relies heavily on glycosylation. It represents a sophisticated evasion mechanism where tumour cells utilise specific glycosylation tags to interfere with macrophage recognition.^15^

CD24 is a glycosylphosphatidylinositol (GPI)‐anchored membrane protein whose functional core is its highly modified glycan structure. It is overexpressed in ovarian cancer and triple‐negative breast cancer, correlating with stemness and poor prognosis.^15^, ^161^, ^162^ Its ligand, Siglec‐10, is an inhibitory receptor on myeloid cells that specifically recognises sialylated glycans on CD24. This interaction transmits a strong inhibitory signal that blocks the initiation of phagocytosis.^15^ This pathway works alongside CD47 and PD‐L1 to allow tumour cells to camouflage as ‘self’.^118^ Notably, some hepatocellular carcinoma sub‐clones co‐express CD24, CD47 and ICAM1, forming a synergistic immune shield.^161^

Intervention strategies focus on relieving this glycosylation‐mediated inhibition. Direct blockade with monoclonal antibodies has shown anti‐tumour effects in preclinical models.^15^ Innovative strategies include bispecific inhibitors (e.g., PAC‐SABI) targeting both CD24 and CD47,^163^ or CD24‐targeted CAR‐T cells that both kill tumour cells and reprogram macrophages towards an M1‐like phenotype.^164^ Other approaches include selectively degrading surface CD24 using LYTACs^165^ or downregulating its expression with DYRK1B inhibitors.^166^ A dual‐function D‐peptide has also been designed to simultaneously block the CD24/Siglec‐10 and PD‐1/PD‐L1 interactions.^167^

#### 3.2.4 ‘Camouflage’ signals: The functional inversion of ICAM‐3 and the hijacking of the DC‐SIGN axis

Tumour cells can use metabolic reprogramming to completely remodel or even reverse the functional instructions of physiological immune recognition signals. ICAM‐3 (CD50) is a prime example of a molecule that has undergone a functional ‘inversion’ from an ‘eat‐me’ signal to a ‘don't‐eat‐me’ signal.

Physiologically, ICAM‐3 is a key adhesion molecule and a classic ‘eat‐me’ signal on apoptotic neutrophils recognised by macrophage LFA‐1 (integrin αLβ2), ensuring safe clearance.^168^ However, in follicular lymphoma and DLBCL, this instruction is subverted.^29^ Somatic mutations introduce new N‐glycosylation sites in the B‐cell receptor (BCR) variable region, leading to the abnormal exposure of high‐mannose structures.^28^ Driven by metabolic disturbances like ER stress, this abnormal glycosylation transforms the ICAM‐3/BCR complex into a ‘metabolic‐derived epitope’.

The core of this functional inversion is the switch in recognition receptors. In the TME, macrophages expressing the C‐type lectin receptor DC‐SIGN bind these mannosylated ligands with high affinity, bypassing the pro‐phagocytic LFA‐1 pathway.^169^ Instead of initiating phagocytosis, this binding transmits BCR‐like survival signals back to the tumour cell and promotes an immunosuppressive microenvironment.^28^ Thus, tumour cells use metabolic abnormalities to create a ‘glycosylation bait’ that hijacks DC‐SIGN, reversing a ‘clearance’ instruction into a ‘support growth’ path.

This mechanism also exists in solid tumours; for example, DC‐SIGN+ TAM infiltration correlates with T cell exhaustion and poor prognosis in bladder and gastric cancers.^170^, ^171^ Blocking DC‐SIGN can restore T cell function and enhance immune checkpoint blockade.^170^ Therapeutic strategies include using small‐molecule inhibitors to block DC‐SIGN's sugar‐recognition function^172^, ^173^ or using mannosylated nanocarriers and DC‐SIGN nanobody‐conjugated vaccines to target antigen‐presenting cells for immune activation.^174^, ^175^

### 3.3 Summary: A new therapeutic paradigm integrating recognition networks and metabolic reprogramming

The recognition of apoptotic tumour cells by macrophages is a precise switch for the immune fate of the tumour. Tumour cells achieve immune escape by systematically hijacking this program, a process rooted in their metabolic abnormalities.^28^, ^103^, ^138^

From a metabolic perspective, tumour cells actively create, modify and amplify surface signals by remodelling lipid, carbohydrate and protein homeostasis.^29^, ^33^, ^139^ Abnormal glycosylation, oxidised lipid modifications and ER stress products serve as ‘abnormal metabolic tags’ that twist clearance signals into instructions for survival and immunosuppression.^15^, ^28^, ^147^ Simultaneously, macrophage receptors such as PS receptors, CD36, Siglec‐10 and DC‐SIGN are ‘tamed’ into sensors for these abnormal tags, driving M2 polarisation and inhibiting interferon production.^16^, ^30^, ^33^

Successful therapeutic strategies must move beyond single‐pathway blockade towards systemic intervention of the metabolic–signalling–recognition–function axis.^118^, ^161^ Future paradigms will focus on:

1. Combinatorial blockade of multiple checkpoints: Overcoming network redundancy by simultaneously targeting axes like CD47‐SIRPα, PD‐1/PD‐L1, CD24‐Siglec‐10 and ICAM‐3‐DC‐SIGN.^15^, ^29^, ^38^, ^163^, ^172^, ^173^
2. Functional conversion and reverse engineering: Transforming evasion mechanisms into therapeutic windows. This includes using mannosylated nanocarriers to deliver TLR agonists to DC‐SIGN‐high TAMs for pro‐inflammatory reprogramming,^174^ or developing bispecific CAR‐T cells targeting CD24 to block ‘don't‐eat‐me’ signals while killing tumour cells.^164^
3. Source metabolic intervention: Reducing the production of immunosuppressive signals by correcting abnormal metabolic states.^161^ Examples include using DYRK1B inhibitors to downregulate CD24,^166^ targeting glycosyltransferases to reverse abnormal glycosylation,^28^ or using YAP inhibitors to modulate redox homeostasis in CD31‐low tumours.^156^
4. Intelligent spatiotemporal delivery systems: Implementing local and dynamic remodelling of immune cell functions in the TME using responsive nanoplatforms,^153^ extracellular vesicle carriers,^138^ or implantable hydrogel systems.^135^

**FIGURE 3:**
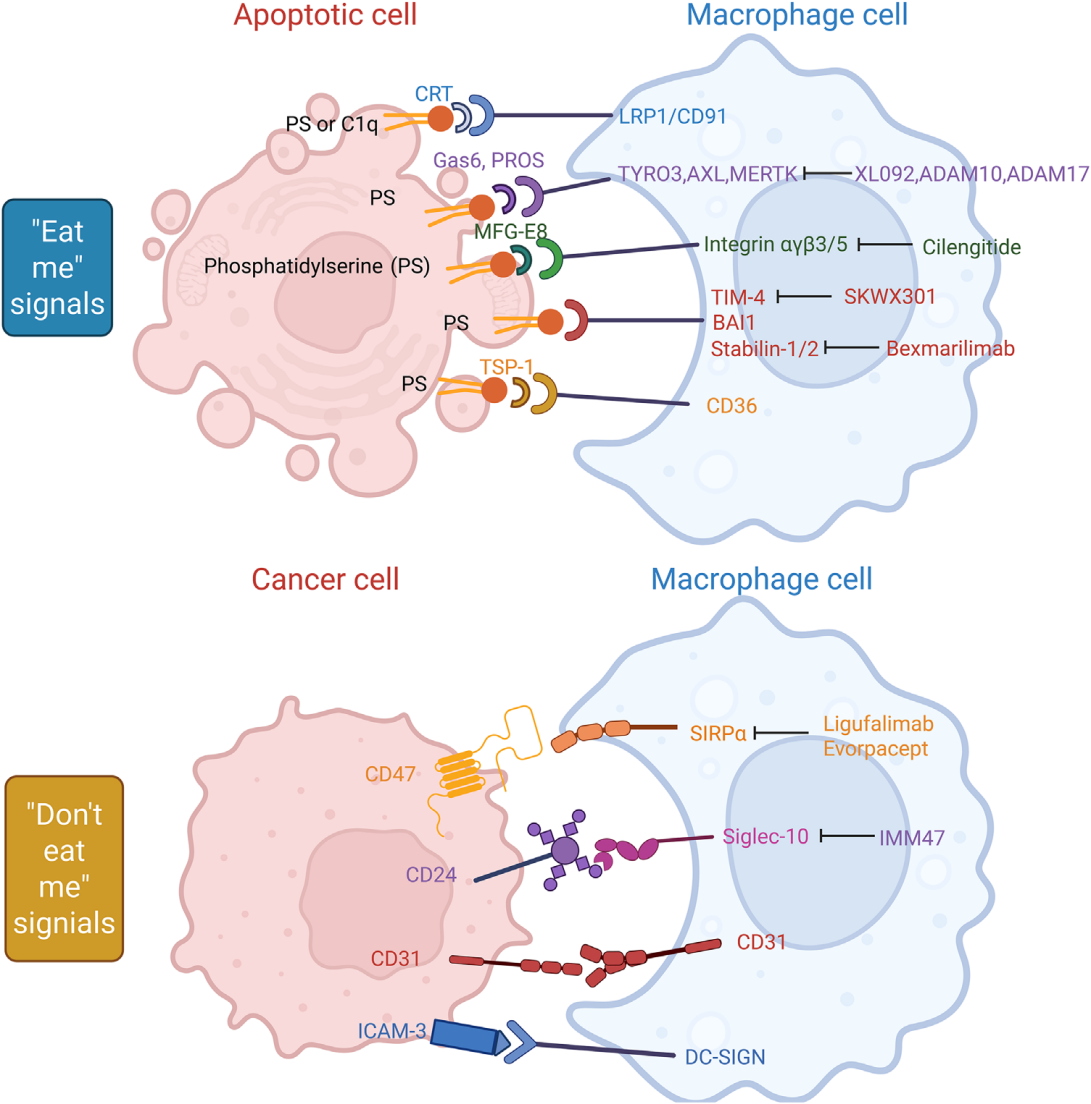
‘Eat‐me’ and ‘don't‐eat‐me’ signals regulating efferocytosis in the tumour microenvironment. Upper panel: Apoptotic tumour cells expose various ‘eat‐me’ signals to facilitate recognition and engulfment by macrophages. Phosphatidylserine (PS) acts as the central ligand, bridging to macrophage receptors (such as TYRO3, AXL, MERTK, Integrin αvβ3/5, TIM‐4, BAI1 and Stabilin‐1/2) via soluble opsonins including Gas6, PROS and MFG‐E8. Other surface ligands like CRT, TSP‐1 and C1q further enhance this interaction through receptors like LRP1/CD91 and CD36. Key therapeutic agents, such as Bexmarilimab (targeting Stabilin‐1/2) and Cilengitide (targeting Integrin αvβ3/5), aim to modulate these pathways to enhance phagocytosis. Lower panel: To evade immune clearance, cancer cells upregulate ‘don't‐eat‐me’ signals, including CD47, CD24, CD31 and ICAM‐3. These ligands engage inhibitory receptors on macrophages, such as SIRPα, Siglec‐10, CD31 and DC‐SIGN, effectively suppressing phagocytic activity. Emerging immunotherapies, including Ligufalimab/Evorpacept (targeting SIRPα) and IMM47 (targeting Siglec‐10), are designed to disrupt these inhibitory axes, thereby restoring anti‐tumour phagocytosis and promoting a pro‐inflammatory tumour immune microenvironment. BAI1, brain‐specific angiogenesis inhibitor 1; CD31, platelet endothelial cell adhesion molecule 1; CD36/47/24, cluster of differentiation 36/47/24; C1q, complement component 1q; CRT, calreticulin; DC‐SIGN, dendritic cell‐specific ICAM‐3‐grabbing non‐integrin; Gas6, growth arrest–specific protein 6; ICAM‐3, intercellular adhesion molecule 3; LRP1/CD91, low‐density lipoprotein receptor‐related protein 1; MFG‐E8, milk fat globule‐EGF factor 8; PROS, protein S; PS, phosphatidylserine; Siglec‐10, sialic acid–binding Ig‐like lectin 10; SIRPα, signal regulatory protein alpha; Stabilin‐1/2, scavenger receptor‐1/2; TIM‐4, T‐cell immunoglobulin and mucin‐domain containing‐4; TSP‐1, thrombospondin‐1; TYRO3/AXL/MERTK (TAM receptors);.

By integrating tumour metabolic abnormalities with immune recognition networks,^104^ particularly at nodes like the ICAM‐3/DC‐SIGN axis,^29^ we can reprogram this hijacked system to release the latent anti‐tumour immune potential of the microenvironment (Figure 3).

## 4 POST‐PHAGOCYTIC DIGESTION AND METABOLIC REPROGRAMMING IN TAMS

In the TME, the clearance of apoptotic cells by macrophages undergoes a fundamental subversion. This homeostatic program, originally designed to terminate inflammation and promote repair, is ‘hijacked’ and remodelled by tumours into a core malignant cycle that drives survival, growth and immune escape.^6^ Its pro‐tumourigenic effects are achieved through three synergistic mechanisms:

1. Sustained immunosuppression: Continuous efferocytosis stabilises TAMs in an M2‐like anti‐inflammatory phenotype, secreting inhibitory mediators such as IL‐10 and TGF‐β. This process can upregulate PD‐L1 and IDO via the activation of the AIM2 inflammasome pathway, leading to long‐term inhibition of cytotoxic T cell and NK cell functions and consolidating the immunosuppressive niche.^176^
2. Metabolic provision and parasitism: Apoptotic cells serve as ‘nutrient packets’, whose catabolism provides TAMs and the entire TME with abundant metabolic substrates, including amino acids, lipids and nucleotides.^177^ These substances are not only utilised to sustain the survival and function of TAMs in harsh conditions like hypoxia^178^ but are also exported to nourish surrounding cancer cells, establishing a parasitic metabolic relationship where ‘tumour cell death fuels tumour cell proliferation’.^32^
3. Epigenetic reprogramming: Specific metabolites from apoptotic cells, such as methionine, drive lasting epigenetic alterations in macrophages. Through mechanisms involving DNMT3A, these changes ‘lock in’ pro‐tumourigenic transcriptional programs, ensuring that macrophages maintain a long‐term immunosuppressive and tissue‐remodelling phenotype, forming a durable ‘metabolic memory’.^32^

Consequently, efferocytosis in tumours has evolved from a simple ‘scavenger’ function into a potent initiating signal that orchestrates immunosuppression, nutrient cycling and the formation of a pro‐metastatic microenvironment.^176^ Upon engulfing apoptotic cells, the metabolic network of macrophages is comprehensively reshaped.^179^ Critically, this reprogramming is not merely a passive result of nutrient influx; apoptotic cells actively release metabolite signals to proactively shape the microenvironment.^8^

### 4.1 The dual complexity of metabolic reprogramming in the TME

Against the backdrop where cancer cells themselves undergo extensive metabolic reprogramming,^180^ the metabolic remodelling of TAMs exhibits a dual complexity: it is intrinsically pro‐repair but is hijacked in the aberrant TME to support tumour growth and consolidate immune tolerance.^7^ This metabolic hijacking provides a critical theoretical basis for combinatorial therapeutic strategies targeting the ‘tumour‐immune’ metabolic crosstalk.^7^

The analysis of Phase III indicates that post‐phagocytic digestion and metabolic reprogramming are key links in the tumour's transformation of efferocytosis from a ‘terminator’ into an ‘enabler’. By utilising apoptotic cells as metabolic and signalling sources, the tumour systematically reprograms the function and fate of macrophages, converting them from potential immune sentinels into collaborators in tumour growth and immune escape. Therefore, intervening in this stage of metabolic reprogramming is a vital strategy to break the ‘apoptosis–efferocytosis–pro‐tumour’ malignant cycle and remodel the anti‐tumour immune microenvironment.

### 4.2 Amino acid metabolism and immunosuppressive polarisation

In the TME, the polarisation of TAMs towards an immunosuppressive phenotype (M2‐like) following efferocytosis profoundly depends on the systematic hijacking and reprogramming of their amino acid metabolic networks. This process is a malignant distortion of sophisticated metabolic programs used in homeostatic repair, primarily involving the arginine, tryptophan and methionine axes. Together, they form a malignant metabolic cycle that drives sustained efferocytosis, self‐reinforcing immunosuppression and ultimate tumour growth.

#### 4.2.1 Arginine–polyamine axis: From repair engine to pro‐tumour driver

In tissue repair, macrophages metabolise arginine from apoptotic cells via the Arginase‐1 (Arg1) pathway to generate ornithine and the polyamine putrescine, which is essential for promoting sustained efferocytosis and inflammatory resolution.^19^ In the TME, this reparative flux is hijacked and amplified. TAMs utilise Arg1 and ornithine decarboxylase (ODC) to convert vast amounts of arginine from apoptotic tumour cells into putrescine. Putrescine not only enhances sustained efferocytic capacity by stabilising the mRNA of the signalling molecule Rac1, forming a self‐sustaining positive feedback loop,^19^ but its downstream polyamines (e.g., spermidine) also modify the translation factor eIF5A via hypusination. This modification is crucial for the efficient translation of a group of key mRNAs, including HIF‐1α.^181^ The upregulation of HIF‐1α translation subsequently drives the glycolytic reprogramming of TAMs and reinforces their M2‐like pro‐tumourigenic transcriptional program. Simultaneously, the exogenous polyamine uptake promoted by efferocytosis directly inhibits the production of pro‐inflammatory cytokines such as IL‐1β, synergistically strengthening immunosuppressive reprogramming.^182^

#### 4.2.2 Tryptophan–kynurenine axis: Constructing a deep immunosuppressive barrier

Tryptophan metabolism is another central hub connecting efferocytosis with immunoregulation. Efferocytosis upregulates indoleamine 2,3‐dioxygenase 1 (IDO1), converting tryptophan to kynurenine, which activates the AhR signalling pathway.^31^ In tumours, this pathway is persistently activated, leading to pathological outcomes. AhR activation not only drives the expression of potent immunosuppressive mediators like IL‐10 and TGF‐β but also further enhances the phagocytic capacity of macrophages via Rac1 activation, forming a positive feedback loop that maintains an immunosuppressive state. Crucially, in the tumour context, this pathway directly mediates therapeutic resistance. Research has shown that macrophages undergoing antibody‐dependent cellular phagocytosis (ADCP) upregulate IDO1 and PD‐L1 by activating the AIM2 inflammasome pathway, thereby inhibiting the killing functions of NK and T cells and leading to the failure of anti‐HER2 therapies.^176^

#### 4.2.3 Glutamine metabolism: Reshaping the energy supply chain

To meet the high energy demands and oxidative stress associated with sustained efferocytosis, TAMs must restructure their energy metabolism. Unlike classically activated inflammatory macrophages, efferocytic macrophages rely on a non‐canonical transamination pathway mediated by glutaminase 1 (Gls1).^183^ This pathway is central to: (1) providing continuous bioenergy for phagocytic activities by supporting mitochondrial oxidative phosphorylation and ATP generation; and (2) maintaining glutathione synthesis to counteract the oxidative stress generated during engulfment, ensuring cell survival and function.^183^

#### 4.2.4 Methionine–DNMT3A axis: Epigenetically ‘locking’ the pro‐tumour program

The most profound manifestation of the hijacking of repair programs is the epigenetic reprogramming mediated by the methionine–DNMT3A axis. Methionine provided by apoptotic cells is converted to S‐adenosylmethionine (SAM) in TAMs and utilised by DNA methyltransferase 3A (DNMT3A) to methylate specific genes.^32^ During repair, this mechanism transiently inhibits the ERK phosphatase DUSP4 to prolong pro‐repair signals. However, in tumours, the continuous supply of apoptotic cells results in a persistent methionine/SAM flux, transforming DNMT3A‐mediated methylation from a transient regulation into a permanent alteration. This leads to the long‐term silencing of multiple negative feedback regulators, including Dusp4, resulting in the chronic abnormal activation of growth‐promoting pathways like ERK and permanently distorting repair factors like TGF‐β1 into mediators of immunosuppression, angiogenesis and metastasis.^32^ This marks the functional transition of TAMs from a plastic state to an epigenetically ‘fixed’ pro‐tumourigenic phenotype.

### 4.3 Lipid metabolism and storage in efferocytic TAMs

In the TME, lipid metabolic reprogramming driven by efferocytosis is the core engine for shaping and maintaining the pro‐tumourigenic state of TAMs. This process not only involves the processing of lipids from apoptotic cells but also profoundly hijacks inherent macrophage metabolic programs, constructing a malignant cycle from ‘lipid‐fuelled tumours’ to ‘lipid‐driven immunosuppressive polarisation’. Its causal network is reflected in three interconnected levels:

1. Driving immunosuppressive polarisation: The core role of the FAO–PPARγ axis following efferocytosis, TAMs undergo a significant metabolic switch, with their primary energy source shifting from glycolysis to FAO.^33^ This transition is not merely for energy; it is a key signal driving functional polarisation. Enhanced FAO activates the JAK1‐STAT6 signalling pathway, directly driving the transcriptional program for M2‐like pro‐tumourigenic genes.^33^ The central molecular hub of this process is the S100A4–CD36–PPARγ axis.^42^
2. Constructing the metabolic provision cycle: Lipid uptake, storage, and export TAMs become lipid‐laden cells by engulfing vast amounts of apoptotic tumour debris and actively export these lipids to nourish the tumour. In glioblastoma, TAMs engulf cholesterol‐rich myelin debris and actively ‘feed’ these lipids to rapidly proliferating mesenchymal‐like tumour cells through an LXR/ABCA1‐dependent pathway, forming a direct ‘metabolic parasitism’.^34^
3. Integration of environmental signals and emerging regulatory nodes stress signals in the TME are deeply integrated into lipid metabolic reprogramming. Hypoxia activates the IRE1–XBP1 branch of the ER stress pathway in TAMs, inhibiting glycolysis while promoting OXPHOS and lipid accumulation.^187^ Additionally, the lipid sensor TREM2 plays a critical role in shaping the immunosuppressive niche by promoting lipid uptake and processing.^184^, ^185^, ^186^, ^187^

### 4.4 Glycolysis and lactate metabolism: The metabolic hub of sustained phagocytosis and immunosuppression

1. Unique glycolytic reprogramming: A dedicated program for sustained engulfment distinct from classical inflammation‐driven glycolysis, efferocytosis induces a unique glycolytic program to support repair activities.^188^ In the TME, the key product lactate upregulates the expression of phagocytic receptors MerTK and LRP1 on the cell surface via calcium‐dependent mechanisms, directly facilitating subsequent rounds of engulfment.^188^
2. The dual function of lactate: A molecular hub connecting energy and immunity intracellular lactate accumulation drives a novel post‐translational modification—histone lactylation. High levels of histone lactylation directly act on the promoters of key immunosuppressive genes like Arg1, enhancing their transcription.^35^ This ‘locks in’ the M2‐like phenotype at an epigenetic level.
3. Metabolic redirection under hypoxia under chronic hypoxia, efferocytic TAMs redirect glucose flux towards the non‐canonical pentose phosphate pathway to generate NADPH, maintaining redox homeostasis and supporting rapid phagolysosomal maturation.^178^

### 4.5 Summary: Core metabolic targets in efferocytosis‐driven reprogramming

The TME hijacks efferocytosis to place TAMs within a sophisticated network composed of amino acid, lipid and carbohydrate metabolic reprogramming.^18^, ^32^ This network is not a simple summation of pathways but forms a highly synergistic, self‐reinforcing metabolic ecosystem.^7^

The three major metabolic axes—amino acids, lipids and glycolysis‐lactate—intertwine to form the synergistic driver network of TAMs. The amino acid axis provides signalling instructions and epigenetic fixation^19^, ^31^, ^32^, ^181^, ^182^, ^183^; the lipid axis establishes energy delivery channels and drives M2 polarisation via the FAO‐PPARγ‐STAT6 hub^33^, ^34^, ^42^, ^189^; and the glycolysis–lactate axis provides dedicated power for sustained engulfment while coupling metabolic status with immune function through histone lactylation.^35^, ^188^, ^190^

**FIGURE 4:**
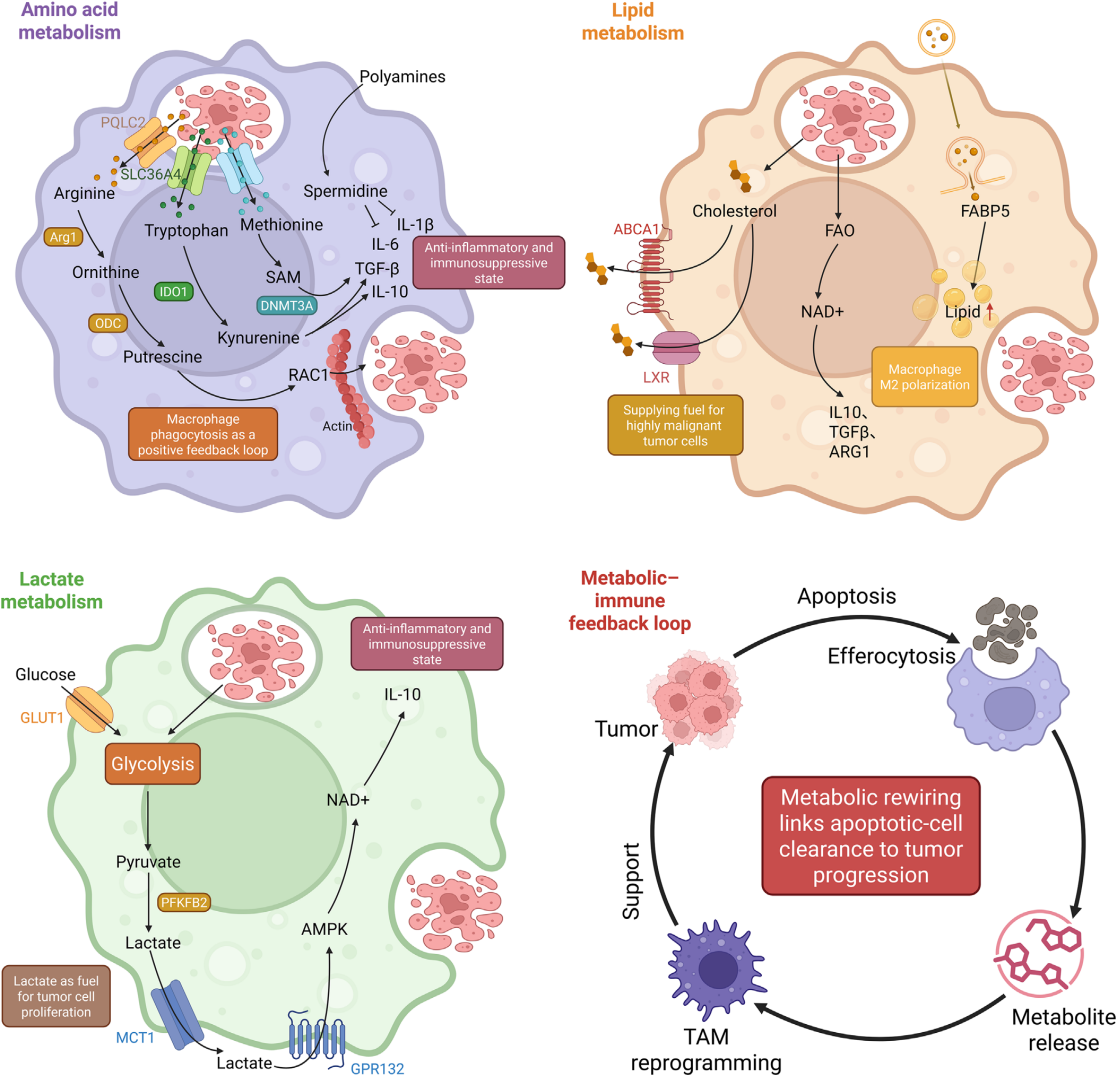
Systematic integration of multi‐axial metabolic reprogramming following efferocytosis. Post‐phagocytic processing of apoptotic debris drives a causal network of metabolic and epigenetic rewiring that solidifies the pro‐tumourigenic landscape. Amino acid metabolism: Phagocytosed apoptotic cells provide rich substrates for amino acid pathways. The arginine–ornithine–putrescine axis (mediated by Arg1 and ODC) establishes a positive feedback loop for sustained engulfment via Rac1 signalling. Concurrently, the methionine–SAM–DNMT3A axis mediates DNA methylation, providing the molecular basis for ‘metabolic memory’ by epigenetically locking macrophages into a persistent M2‐like immunosuppressive state. The tryptophan–kynurenine axis (via IDO1) further activates the AhR pathway to reinforce immune tolerance. Lipid metabolism: Engulfed lipids and cholesterol activate the LXR and fatty acid oxidation (FAO) pathways, shifting energy metabolism and driving M2 polarisation through NAD+‐dependent signalling and anti‐inflammatory cytokine induction (IL‐10, TGF‐β). Notably, accumulated lipids are not merely stored but are reverse‐transported via ABCA1 to establish ‘nutrient fuelling’ of neighbouring malignant cells, creating a symbiotic parasitic relationship between dying cells and proliferating survivors. Lactate metabolism: Efferocytosis induces a unique glycolytic program (mediated by PFKFB2) leading to massive lactate production and secretion via MCT1. Extracellular lactate serves as an alternative fuel for tumour cells while intracellularly inducing histone lactylation, which directly activates the transcription of immunosuppressive genes like Arg1. Furthermore, lactate signals back via GPR132 to enhance AMPK activation, further promoting an anti‐inflammatory state. Metabolic–immune feedback loop: The final panel integrates these pathways into a self‐reinforcing cycle. Apoptosis triggers efferocytosis, which releases metabolites that simultaneously reprogram TAMs and directly support tumour growth. This holistic hijacking ensures the transition from transient tissue repair to chronic malignant progression. ABCA1, ATP‐binding cassette transporter A1; AMPK, AMP‐activated protein kinase; Arg1, arginase 1; ARG1, arginase 1; DNMT3A, DNA (cytosine‐5)‐methyltransferase 3A; FABP5, fatty acid‐binding protein 5; FAO, fatty acid oxidation; GLUT1, glucose transporter type 1; GPR132, G protein‐coupled receptor 132; IDO1, indoleamine 2,3‐dioxygenase 1; IL‐10, interleukin‐10; LXR, liver X receptor; MCT1, monocarboxylate transporter 1; NAD^+^, nicotinamide adenine dinucleotide; ODC, ornithine decarboxylase; PFKFB2, 6‐phosphofructo‐2‐kinase/fructose‐2,6‐biphosphatase 2; PQLC2, PQ loop repeat–containing protein 2; RAC1, Ras‐related C3 botulinum toxin substrate 1; SAM, S‐adenosylmethionine; SLC36A4, solute carrier family 36 member 4; TAM, tumour‐associated macrophage; TGF‐β, transforming growth factor beta.

Interfering with this integrated metabolic network is key to breaking the malignant cycle. Future therapeutic strategies must focus on the holism of this metabolic network.^7^, ^18^ Single‐pathway interventions may fail due to metabolic redundancy; however, combinatorial targeting of key nodes across different axes, or integrating them with immune checkpoint blockade, holds promise for fundamentally dismantling the pro‐tumourigenic functions of TAMs and reversing the immunosuppressive microenvironment (Figure 4).

## 5 THERAPEUTIC TARGETING OF EFFEROCYTOSIS IN THE TUMOUR MICROENVIRONMENT

In summary, the clearance of apoptotic cells within the TME has evolved from a homeostatic physiological process into a pro‐tumourigenic malignant cycle driven by systemic metabolic reprogramming.^6^ This axis spans from the release of metabolic instructions by apoptotic cells and the decoding of surface recognition signals to profound post‐phagocytic metabolic rewiring. The preceding sections of this review have systematically elucidated the core mechanisms of these three stages, emphasising the pivotal role of metabolic reprogramming in driving functional inversion.^7^ Notably, these mechanisms are not isolated; they have been rigorously validated through a series of precise gene knockout and functional intervention models, which clarify the specific roles of key signalling molecules and metabolic pathways. These studies not only solidify the theoretical foundation but also provide clear molecular targets and intervention logics for developing therapeutic strategies targeting this axis.^117^

Given that efferocytosis has been hijacked as an engine for immunosuppression and metabolic fuelling in cancer, intervening in this process represents a potential breakthrough for remodelling the tumour immune microenvironment and overcoming therapeutic resistance. In recent years, based on a deeper understanding of ‘find‐me’, ‘eat‐me’ and ‘don't‐eat‐me’ signalling pathways, as well as the identification of post‐phagocytic metabolic reprogramming nodes, various therapeutic strategies targeting different stages of efferocytosis have emerged. These strategies encompass small‐molecule inhibitors, monoclonal antibodies, fusion proteins, engineered cell therapies and metabolic modulators, aiming to transform immunosuppressive death clearance into an anti‐tumour immune activation event by blocking harmful signals, enhancing beneficial recognition or reprogramming macrophage functions. This chapter will systematically review representative therapeutic strategies currently in preclinical and clinical development, discussing their mechanisms of action, preclinical evidence, clinical progress and challenges, providing a reference for future precision immunometabolic therapies targeting efferocytosis.

### 5.1 Gene knockout and functional intervention models: Systematically validating the specificity of metabolic signals driving efferocytosis and immunosuppression

Gene knockout and functional intervention studies of key targets in the efferocytic process provide the most direct experimental evidence for understanding their specific mechanisms within the TME. These models systematically reveal how metabolic signals drive macrophage recruitment, recognition and metabolic reprogramming, thereby promoting tumour immunosuppression and malignant progression. The following sections elaborate on the findings and significance of key gene knockout and intervention models across the ‘find‐me’, ‘eat‐me’ and ‘metabolic reprogramming’ stages.

In the ‘find‐me’ signalling stage, multiple studies using gene knockout models have validated the central role of specific metabolic signals in regulating macrophage recruitment. Nucleotides (ATP/UTP) released by apoptotic cells are classic ‘find‐me’ signals, whose release mechanism depends on the activation of the Panx1 channel. Chekeni et al. found that knocking out Panx1 or inhibiting its function significantly reduces nucleotide release and impairs the chemotactic recruitment of monocytes/macrophages.^11^ Similarly, the P2Y2 receptor acts as a key sensor for ATP/UTP; in P2Y2‐deficient mouse models, the clearance efficiency of apoptotic thymocytes is significantly decreased, confirming the necessity of purinergic signalling in in vivo efferocytosis.^45^ Regarding lipid signals, ABCA1‐mediated LPC release is a critical step for recruiting phagocytes, and its knockout completely abolishes LPC release and monocyte chemotaxis.^50^ Sphingosine kinases (SphKs) also play a core role in regulating S1P production: knockdown of SphK2 (but not SphK1) in tumour cells reverses apoptosis‐induced M2 polarisation and restores pro‐inflammatory responses.^55^ Furthermore, Spns2 knockout mice exhibit reduced blood S1P levels, lymphocyte migration defects and lymphatic structure abnormalities, further confirming the systemic role of S1P signalling in immune cell trafficking and microenvironmental shaping.^191^

In the recognition stage of ‘eat‐me’ and ‘don't‐eat‐me’ signals, gene knockout models have revealed the specific functions of key receptors in regulating phagocytic efficiency and immunological outcomes. PS is the core ‘eat‐me’ signal, whose exposure is regulated by the dynamic balance between scramblases and flippases; knockout of the flippase ATP11C enhances the PS exposure phenotype, confirming that PS externalisation results from an equilibrium between activating and inhibitory signals.^101^ On the macrophage side, knockdown of Stabilin‐1 (STAB1) in AML models reduces tumour cell proliferation and reverses M2 polarisation by inhibiting the IKK/NF‐κB pathway.^30^ As a phagocytic receptor for oxPL, CD36 knockout mice are protected from oxPL‐induced lung fibrosis, with significantly reduced oxPL accumulation and TGF‐β expression in macrophages, highlighting CD36's role in linking lipid sensing to pro‐fibrotic responses.^68^ Regarding ‘don't‐eat‐me’ signals, many tumours overexpress CD24 while TAMs express high levels of Siglec‐10; either knocking out CD24/Siglec‐10 or using antibodies to block their interaction significantly enhances the phagocytosis of CD24‐expressing ovarian and triple‐negative breast cancer cells.^15^ CD47 knockout or antibody blockade restores macrophage phagocytosis, which can be synergistically enhanced when combined with agents inducing CRT exposure.^39^

In the metabolic reprogramming stage, gene knockout and intervention models have systematically revealed the causal roles of amino acid, lipid and glucose metabolic axes in driving the pro‐tumourigenic phenotype of macrophages. Arginine metabolism is key to polyamine synthesis; knockout of Arg1 or DNMT3A in macrophages blocks epigenetic reprogramming mediated by apoptotic cell‐derived methionine, thereby inhibiting the expression of pro‐repair mediators (e.g., PGE2, TGF‐β), impairing tissue repair and delaying tumour progression.^32^ In lipid metabolism, S100A4‐knockout macrophages fail to induce PPAR‐γ‐dependent FAO, leading to weakened pro‐tumour polarisation.^42^ As the rate‐limiting enzyme of FAO, epithelial‐specific knockout of CPT1A in mice enhances tumour sensitivity to ferroptosis and reverses TAM‐mediated CD8+ T cell suppression, thus sensitising tumours to immunotherapy.^71^ In the glycolysis–lactate axis, genetic and pharmacological inhibition of LDHA‐mediated tumour‐macrophage symbiosis significantly suppresses tumour progression and macrophage infiltration in glioblastoma models.^78^ Additionally, in pancreatic cancer liver metastasis models, macrophage‐specific knockout of Progranulin (PGRN) blocks lysosomal acidification and phagocytic degradation, thereby inhibiting Arg1 upregulation and pro‐metastatic transformation, reducing liver metastasis and improving CD8+ T cell function.^192^

**FIGURE 5:**
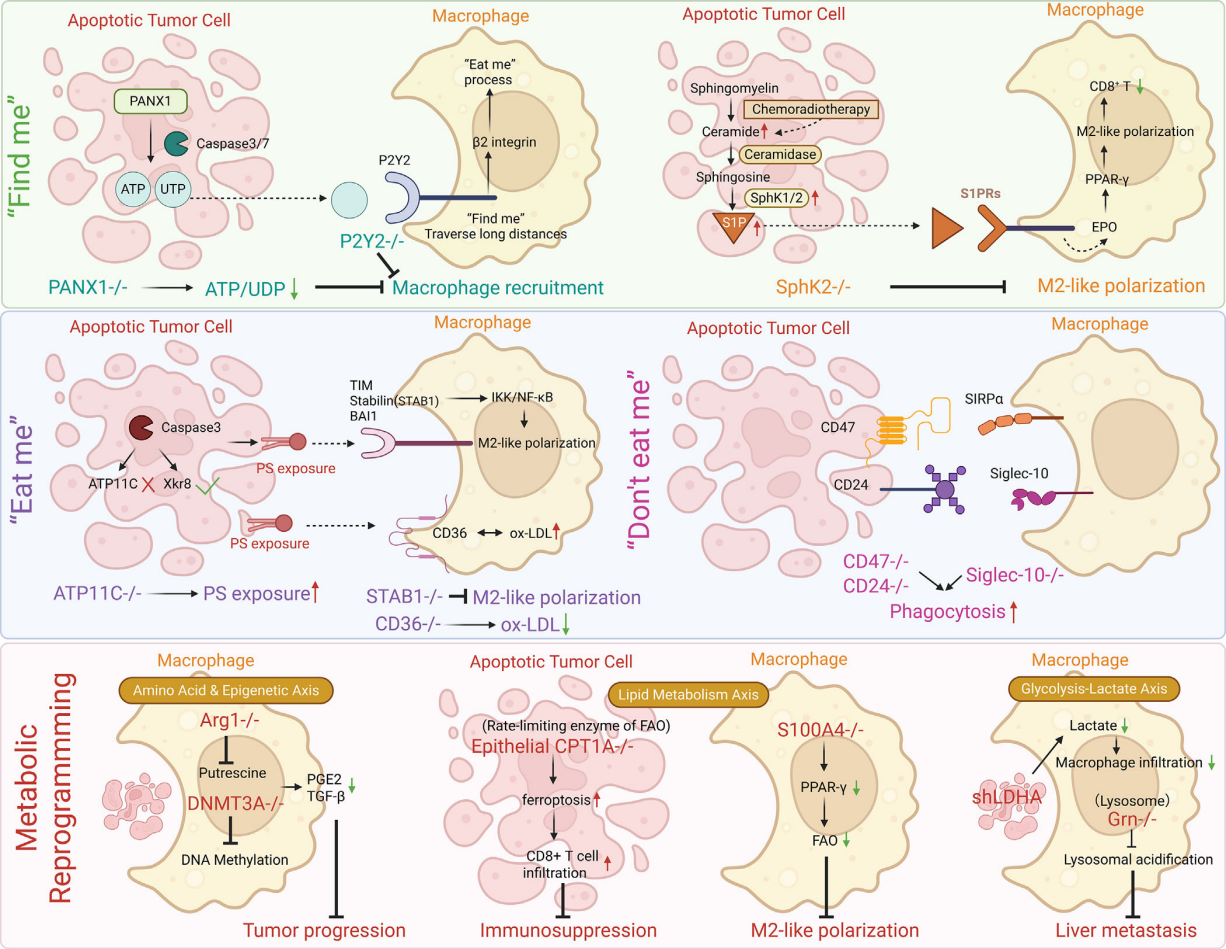
Systematic validation of the functional specificity and therapeutic potential of efferocytosis‐related nodes. This integrated schematic illustrates the multi‐stage regulation of apoptotic‐cell clearance and the causal role of metabolic reprogramming in driving the pro‐tumourigenic landscape. Top (find‐me stage): Genetic ablation of PANX1 or P2Y2 (indicated by red crosses) disrupts the nucleotide‐mediated recruitment axis, demonstrating the necessity of ATP/UTP signalling for macrophage mobilisation. Middle (eat‐me and don't‐eat‐me stage): The specificity of surface recognition is validated by knockout models of key receptors and checkpoints. Deficiency in STAB1 or CD36 impairs anti‐inflammatory signalling and lipid sensing, respectively, while the genetic or pharmacological blockade of the CD24–Siglec‐10 or CD47–SIRPα axis (the ‘don't‐eat‐me’ signals) restores the phagocytic ‘brakes’, enabling macrophages to attack malignant cells. Bottom (metabolic reprogramming): Causal links between metabolism and macrophage phenotype are established through targeted interventions. In the amino acid axis, knockout of Arg1 or DNMT3A prevents epigenetic ‘locking’ of immunosuppressive programs. In lipid metabolism, S100A4 deficiency impairs PPAR‐γ‐dependent fatty acid oxidation (FAO). In the glycolysis–lactate axis, epithelial CPT1A deletion sensitises tumours to ferroptosis and restores CD8^+^ T‐cell infiltration, while Progranulin knockout blocks lysosomal acidification to inhibit macrophage‐mediated metastasis. Collectively, these models provide definitive evidence that targeting these molecular nodes can systematically reprogram efferocytic.

In conclusion, gene knockout and functional intervention models provide indispensable evidence for validating the specific roles of metabolic signals during efferocytosis. These models not only confirm the necessity of ‘find‐me’, ‘eat‐me’ and ‘don't‐eat‐me’ signals in regulating macrophage recruitment and recognition but also reveal the central position of metabolic reprogramming in driving the pro‐tumourigenic phenotype of macrophages. Future research should further integrate cell‐specific knockouts, spatiotemporally controllable gene editing and multi‐omics analysis to more precisely dissect the crosstalk between different metabolic axes in the TME, providing a theoretical basis for developing precision metabolic‐immunotherapy strategies (Figure 5).

### 5.2 Targeting the ‘find‐me’ stage

The initial ‘find‐me’ stage offers a strategic opportunity to intercept the efferocytic process at its source. This stage is regulated by chemotactic metabolites including nucleotides (ATP, UTP), lipid mediators (S1P, LPA, LPC, PGE2, oxPLs), polyamines and lactate. Therapeutic strategies aim to neutralise these signals or block corresponding receptors to prevent the recruitment and initial activation of pro‐tumour macrophages. Current clinical research focuses heavily on S1P. Representative agents include suramin, fingolimod (FTY720) and sonepcizumab (LT1009), which are at various stages of clinical evaluation (Table 1).

**TABLE 1:** Pharmacological modulation of sphingosine‐1‐phosphate (S1P) signalling in the ‘find‐me’ stage of apoptotic‐cell clearance and tumour immunoregulation.

Drug name/target	NCT number	Tumour type treated	Status	Phases	Study type
Sonepcizumab (S1P mAb)	NCT00661414	Advanced solid tumours	Completed	Phase 1	Interventional
Sonepcizumab (S1P mAb)	NCT01762033	Unresectable and refractory renal cell carcinoma	Terminated	Phase 2	Interventional
Fingolimod (S1PR modulator)	NCT03941743	Breast cancer	Completed	Phase 1	Interventional
Fingolimod (S1PR modulator)	NCT06424067	Lung cancers (NSCLC and SCLC)	Recruiting	Phase 2	Interventional
Fingolimod (S1PR modulator)	NCT02490930	High‐grade glioma (glioblastoma, anaplastic astrocytoma)	Completed	Early Phase 1	Interventional
Fingolimod (S1PR modulator)	NCT06705608	Skin cancer in multiple sclerosis patients	Completed	N/A	Observational
Suramin (multi‐kinase/S1PR inhibitor)	NCT00002723	Hormone‐refractory prostate cancer	Completed	Phase 3	Interventional
Suramin (multi‐kinase/S1PR inhibitor)	NCT00001266	Prostate carcinoma	Completed	Phase 2	Interventional
Suramin (multi‐kinase/S1PR inhibitor)	NCT00002652	Multiple myeloma or Castleman's disease	Completed	Phase 2	Interventional
Suramin (multi‐kinase/S1PR inhibitor)	NCT00001381	Superficial bladder cancer	Completed	Phase 1	Interventional
Suramin (multi‐kinase/S1PR inhibitor)	NCT00002639	Recurrent primary brain tumours	Completed	Phase 2	Interventional
Suramin (multi‐kinase/S1PR inhibitor)	NCT00004073	Glioblastoma multiforme	Completed	Phase 2	Interventional
Suramin (multi‐kinase/S1PR inhibitor)	NCT00054028	Stage IIIB–IV breast cancer	Completed	Phase 1/2	Interventional
Suramin (multi‐kinase/S1PR inhibitor)	NCT00006476	Recurrent bladder cancer	Completed	Phase 1	Interventional
Suramin (multi‐kinase/S1PR inhibitor)	NCT00003038	Advanced solid tumours	Completed	Phase 1	Interventional
Suramin (multi‐kinase/S1PR inhibitor)	NCT01671332	Advanced NSCLC	Completed	Phase 2	Interventional
Suramin (multi‐kinase/S1PR inhibitor)	NCT00002921	Stage III or IV adrenocortical cancer	Terminated	Phase 2	Interventional
Suramin (multi‐kinase/S1PR inhibitor)	NCT00083109	Metastatic renal cell cancer	Completed	Phase 1/2	Interventional
Suramin (multi‐kinase/S1PR inhibitor)	NCT01038752	Non‐small cell lung cancer	Terminated	Phase 2	Interventional
Suramin (multi‐kinase/S1PR inhibitor)	NCT00006929	Stage IIIB/IV non‐small cell lung cancer	Completed	Phase 2	Interventional
Suramin (multi‐kinase/S1PR inhibitor)	NCT00066768	Platinum‐refractory NSCLC	Completed	Phase 1	Interventional
Suramin (multi‐kinase/S1PR inhibitor)	NCT00002881	Prostate cancer	Completed	Phase 3	Interventional

#### 5.2.1 Suramin

Suramin is a multifunctional compound whose anticancer activity stems from its properties as a broad‐spectrum multi‐kinase inhibitor and growth factor antagonist.^193^ In the context of efferocytosis, its core mechanism involves antagonising S1P receptors, thereby interfering with the directional migration of macrophages towards apoptotic tumour regions.^193^ Additionally, suramin blocks signalling from growth factors such as aFGF/bFGF, PDGF and TGF‐β.^193^, ^194^ Preclinical studies show its potential as a chemosensitiser; in breast cancer models, low‐dose suramin combined with paclitaxel significantly improved complete response rates without increasing toxicity.^194^, ^195^ However, clinical outcomes have shown significant heterogeneity across tumour types, with positive signals in bladder^196^ and prostate cancer,^197^ but insufficient objective response rates in metastatic breast cancer.^198^ Its broad toxicity profile (e.g., proteinuria, neuroinflammation) highlights the necessity of evolving from ‘broad‐spectrum inhibition’ towards ‘precision targeting’.^193^, ^199^

#### 5.2.2 Fingolimod (FTY720)

Fingolimod acts with higher precision than suramin as an S1P receptor modulator. It functions as a functional antagonist of S1PR1 and inhibits sphingosine kinase activity.^200^, ^201^ In the ‘find‐me’ stage, FTY720 directly blocks macrophage reception of S1P chemotactic signals. Beyond recruitment, it inhibits tumour cell migration, invasion and induction of non‐caspase‐3‐dependent cell death.^202^, ^203^, ^204^ Its clinical value depends on biomarker‐driven precision; for instance, S1PR1 T236 phosphorylation levels may serve as a predictive marker in triple‐negative breast cancer.^202^, ^203^, ^205^

#### 5.2.3 Sonepcizumab (LT1009)

Sonepcizumab represents a more direct and specific pharmacological strategy for targeting ‘find‐me’ signalling. As a humanised monoclonal antibody, it neutralises circulating S1P with high affinity and specificity.^206^ Unlike suramin (receptor antagonism) or fingolimod (receptor modulation), sonepcizumab acts by ‘mopping up’ extracellular S1P ligands, thereby preventing their binding to S1PR1 on macrophages and other immune cells at the source.^206^, ^207^ This mechanism effectively disrupts S1P‐mediated macrophage chemotaxis and activation, theoretically intercepting the recruitment of TAMs to apoptotic regions during the ‘find‐me’ phase. Moreover, since S1P signalling directly promotes angiogenesis and immunosuppression, its neutralisation may yield pleiotropic anti‐tumour effects.

In a Phase II clinical trial for metastatic renal cell carcinoma refractory to VEGF inhibitors, sonepcizumab monotherapy demonstrated a favourable safety profile; the primary adverse events were grade 1–2 fatigue and weight gain, with no grade 3/4 serious adverse events occurring in more than 5% of patients.^207^ However, the study failed to meet its primary endpoint for progression‐free survival (PFS), with an objective response rate (ORR) of only 10%. Notably, the median overall survival (OS) reached 21.7 months, and durable responders frequently harboured VHL and PBRM1 mutations, suggesting potential predictive biomarkers.^207^ These results indicate that as a monotherapy, sonepcizumab's anti‐tumour activity is insufficient to reach the clinical efficacy threshold in unselected populations. The clinical experience with sonepcizumab provides pivotal guidance for the development of ‘find‐me’ signal‐targeted therapies. First, its limited monotherapy efficacy underscores that blocking the S1P signal alone is inadequate to reverse a well‐established and highly complex immunosuppressive TME,^207^ highlighting the necessity of shifting towards combinatorial strategies. Given that the S1P pathway mediates resistance to existing targeted and immunotherapies^207^ and plays key roles in promoting angiogenesis^57^ and suppressing anti‐tumour immunity,^58^ combining sonepcizumab with mechanistically synergistic agents has a solid theoretical foundation. For instance, co‐administration with anti‐angiogenic drugs could simultaneously strike the tumour's blood supply and S1P‐dependent immunosuppressive recruitment. Similarly, combining it with ICIs may weaken innate immunosuppression while releasing the adaptive immune ‘brake’, potentially generating synergistic outcomes.

Second, biomarker‐guided precision medicine is fundamental to realising clinical value. The correlation between VHL/PBRM1 mutations and durable responses observed in RCC^207^ strongly suggests the need to integrate genomics to identify patient subpopulations whose tumour progression is particularly dependent on the S1P axis. Finally, the strategy of targeting the circulating ligand (S1P) offers a unique advantage by avoiding potential desensitisation or redundancy issues associated with receptor targeting. Its demonstrated safety profile validates the feasibility of this strategy,^207^ although its ultimate clinical value must be confirmed through rigorously designed combination clinical trials.^208^

### 5.3 Targeting the ‘eat‐me’ stage

The ‘eat‐me’ stage centres on the precise molecular recognition between phagocytes and apoptotic cells. Therapeutic strategies for this stage are two‐fold: first, marking tumour cells for immune‐mediated destruction via exposed ‘eat‐me’ signals such as PS; and second, blocking the pro‐tumourigenic and immunosuppressive signalling cascades triggered when macrophages recognise these signals through receptors like the TAM family (TYRO3, AXL, MERTK). In the TME, metabolic stressors such as hypoxia and oxidative stress drive the aberrant externalisation of PS on both tumour cells and vascular endothelial cells, providing a widely distributed target for disrupting tumour blood supply and reversing immunosuppression.

#### 5.3.1 Anti‐PS antibodies

Bavituximab represents a ‘functional conversion’ strategy that reprograms the biological outcome of PS recognition. As an IgG1 antibody, its Fc domain recruits effector cells to initiate ADCC against PS‐positive tumour vessels and malignant cells.^209^, ^210^ This converts the ‘silent clearance’ signal of PS into a pro‐inflammatory ‘attack’ signal. Furthermore, Bavituximab directly reverses myeloid polarisation by blocking the tolerance signals transmitted by PS to TAMs and MDSCs, thereby reprogramming macrophages from an M2 to an M1 phenotype.^211^

Preclinical evidence supports combining Bavituximab with radiotherapy or chemotherapy, which significantly increases PS exposure.^209^, ^210^ While Phase I/II trials in NSCLC and breast cancer showed promising safety and efficacy signals,^212^, ^213^ subsequent Phase III trials failed to meet primary endpoints. Critical lessons from these failures include: (1) the narrow therapeutic window due to broad PS exposure across various pathophysiological processes^214^; and (2) the lack of predictive biomarkers to identify PS‐sensitive patient populations.^215^ Future development must prioritise the identification of biomarkers based on PS exposure levels or myeloid characteristics and explore synergistic combinations with ICIs or therapies inducing ICD to fully unlock its immunomodulatory potential.^209^, ^210^

#### 5.3.2 AXL as a hub for phagocytic recognition and therapeutic targeting

Beyond direct PS targeting, inhibiting the macrophage receptor AXL is a core strategy. AXL, a TAM family kinase, binds PS via the bridging molecule Gas6. High‐selectivity inhibitors like Bemcentinib (BGB324/R428) aim to intervene in the post‐phagocytic signalling rather than blocking engulfment itself, thereby avoiding secondary necrosis and inflammation. By inhibiting AXL kinase activity, Bemcentinib blocks the immunosuppressive signals of the AXL–Gas6–PS axis and reprograms TAMs towards an anti‐tumour M1‐like phenotype.^216^ It also downregulates checkpoints like PD‐L1 on both macrophages and tumour cells.^216^

Clinical translation of Bemcentinib relies on its ability to reverse malignant phenotypes such as epithelial–mesenchymal transition (EMT) and acquired resistance to Epidermal Growth Factor Receptor (EGFR)**GPI** inhibitors.^217^, ^218^ Preclinical data across CML, head and neck cancer, and NSCLC confirm its potential to sensitise resistant cells to targeted therapies.^217^, ^218^, ^219^, ^220^ Given the strong correlation between high AXL expression and poor prognosis, establishing AXL expression as a predictive biomarker is crucial for future clinical success.^218^, ^220^, ^221^ Rational combinations with PD‐1/PD‐L1 inhibitors or EGFR inhibitors represent the most promising paths for overcoming therapeutic resistance.^216^, ^217^, ^218^, ^219^

#### 5.3.3 Additional AXL and MERTK targeted agents

A diverse toolkit of agents targeting AXL and MERTK is currently under development. These drugs focus on interfering with hijacked post‐phagocytic pathways to reverse the immunological and metabolic consequences of efferocytosis. For instance, TP‐0903 and ONO‐7475 restore sensitivity to FLT3 or EGFR inhibitors in mutated cancer cells,^222^, ^223^ while the MERTK inhibitor MRX‐2843 reverses resistance to osimertinib in NSCLC.^224^ These agents also modulate TAM polarisation; TP‐0903 blocks STAT6‐driven M2 polarisation,^225^ and MRX‐2843 activates CD8+ T cell immunity by enhancing dendritic cell function.^226^

Emerging strategies also include the soluble AXL decoy receptor Batiraxcept (AVB‐S6‐500), which captures GAS6 to inhibit the PS–GAS6–AXL axis,^227^ and AXL‐targeted antibody‐drug conjugates (ADCs) that specifically deplete AXL‐high tumour cells and M2‐like TAMs.^228^

The clinical potential of these agents lies in a synergistic ‘synchronous strike and ecological remodelling’ mechanism. While cytotoxic therapies generate vast amounts of apoptotic debris that could trigger immunosuppressive efferocytosis, AXL/MERTK inhibitors block this pathological feedback, preventing the formation of an ‘ecological sanctuary’ for residual tumour cells.^223^, ^229^ This environment‐reshaping effect creates a window for ICIs: by clearing or reprogramming M2‐like TAMs and promoting antigen presentation, AXL/MERTK inhibition transforms ‘cold’, immunosuppressive TMEs into ‘hot’ environments receptive to T cell infiltration.^226^, ^228^ Ultimately, by orchestrating the ‘eat‐me’ signalling context, these interventions reprogram tumour cell death from a ‘silent clearance’ into an ‘immunogenic event’ capable of initiating durable anti‐tumour immunity (Table 2).

**TABLE 2:** Summary of representative agents targeting phosphatidylserine (PS) exposure and tumour‐associated macrophage (TAM)family signalling, with mechanisms, key preclinical findings and status of clinical development.

Drug name/target	NCT number	Tumour type treated	Status	Phases	Study type
Bemcentinib (AXL inhibitor)	NCT02424617	Non‐small cell lung cancer	Completed	Phase 1/2	Interventional
Bemcentinib (AXL inhibitor)	NCT03184571	Advanced non‐small cell lung cancer	Completed	Phase 2	Interventional
Bemcentinib (AXL inhibitor)	NCT02922777	Non‐small cell lung cancer	Completed	Phase 1	Interventional
Bemcentinib (AXL inhibitor)	NCT05469178	Advanced/metastatic non‐small cell lung cancer	Terminated	Phase 1/2	Interventional
Bemcentinib (AXL inhibitor)	NCT03184558	Triple‐negative breast cancer	Terminated	Phase 2	Interventional
Bemcentinib (AXL inhibitor)	NCT03649321	Metastatic pancreatic cancer	Terminated	Phase 1/2	Interventional
Bemcentinib (AXL inhibitor)	NCT03965494	Recurrent glioblastoma	Terminated	Early Phase 1	Interventional
Bemcentinib (AXL inhibitor)	NCT02488408	Acute myeloid leukaemia/MDS	Completed	Phase 1/2	Interventional
Bemcentinib (AXL inhibitor)	NCT03824080	Myelodysplastic syndromes/AML	Completed	Phase 2	Interventional
Bemcentinib (AXL inhibitor)	NCT03654833	Malignant mesothelioma	Unknown Status	Phase 2	Interventional
Bavituximab (PS‐targeting mAb)	NCT04099641	Advanced gastric/GEJ cancer	Completed	Phase 2	Interventional
Bavituximab (PS‐targeting mAb)	NCT03519997	Advanced hepatocellular carcinoma	Active, not recruiting	Phase 2	Interventional
Bavituximab (PS‐targeting mAb)	NCT04150900	Head and neck squamous cell carcinoma	Active, not recruiting	Phase 2	Interventional
Bavituximab (PS‐targeting mAb)	NCT01999673	Late‐stage non‐squamous NSCLC	Completed	Phase 3	Interventional
Bavituximab (PS‐targeting mAb)	NCT01138163	Locally advanced/metastatic non‐squamous NSCLC	Completed	Phase 2	Interventional
Bavituximab (PS‐targeting mAb)	NCT01160601	Previously untreated NSCLC	Completed	Phase 2	Interventional
Bavituximab (PS‐targeting mAb)	NCT00687817	Non‐small cell lung cancer	Completed	Phase 2	Interventional
Bavituximab (PS‐targeting mAb)	NCT01323062	Chemo‐Naive Stage IV non‐squamous NSCLC	Completed	Phase 1	Interventional
Bavituximab (PS‐targeting mAb)	NCT02673814	Previously treated metastatic NSCLC	Withdrawn	Phase 2	Interventional
Bavituximab (PS‐targeting mAb)	NCT00669591	Advanced breast cancer	Completed	Phase 2	Interventional
Bavituximab (PS‐targeting mAb)	NCT00669565	Stage IV breast cancer	Completed	Phase 2	Interventional
Bavituximab (PS‐targeting mAb)	NCT01288261	HER2‐negative metastatic breast cancer	Completed	Phase 1	Interventional
Bavituximab (PS‐targeting mAb)	NCT02651610	HER2‐negative metastatic breast cancer	Withdrawn	Phase 2/3	Interventional
Bavituximab (PS‐targeting mAb)	NCT02685306	Early‐stage triple‐negative breast cancer	Withdrawn	Phase 2	Interventional
Bavituximab (PS‐targeting mAb)	NCT01272791	Previously untreated Stage IV pancreatic cancer	Completed	Phase 2	Interventional
Bavituximab (PS‐targeting mAb)	NCT03139916	Newly diagnosed glioblastoma	Completed	Phase 2	Interventional
Bavituximab (PS‐targeting mAb)	NCT01264705	Advanced liver cancer	Completed	Phase 1/2	Interventional
Bavituximab (PS‐targeting mAb)	NCT05249569	Advanced hepatocellular carcinoma	Terminated	Phase 2	Interventional
Bavituximab (PS‐targeting mAb)	NCT02989870	Unresectable hepatocellular carcinoma	Withdrawn	Phase 1	Interventional
Bavituximab (PS‐targeting mAb)	NCT01335204	Castration‐resistant prostate cancer	Terminated	Phase 1/2	Interventional
Bavituximab (PS‐targeting mAb)	NCT01634685	Rectal adenocarcinoma	Completed	Phase 1	Interventional
Bavituximab (PS‐targeting mAb)	NCT01984255	Advanced melanoma	Terminated	Phase 1	Interventional
Bavituximab (PS‐targeting mAb)	NCT00129337	Advanced solid tumours	Completed	Phase 1	Interventional
TP‐0903	NCT02729298	Advanced solid tumours	Completed	Phase 1	Interventional
TP‐0903	NCT03572634	Chronic lymphocytic leukaemia	Terminated	Phase 1/2	Interventional
TP‐0903	NCT04518345	FLT3‐mutated acute myeloid leukaemia	Completed	Early Phase 1	Interventional
Cabozantinib (AXL inhibitor)	NCT01639508	RET fusion‐positive advanced NSCLC	Recruiting	Phase 2	Interventional
Cabozantinib (AXL inhibitor)	NCT03170960	Advanced solid tumours	Active, not recruiting	Phase 1	Interventional
Cabozantinib (AXL inhibitor)	NCT04316182	Hepatocellular carcinoma	Completed	Phase 2	Interventional
Cabozantinib (AXL inhibitor)	NCT03425201	Advanced urothelial cancer	Completed	Phase 1/2	Interventional
Cabozantinib (AXL inhibitor)	NCT04514484	Advanced cancer in patients with HIV	Active, not recruiting	Phase 1	Interventional
Cabozantinib (AXL inhibitor)	NCT03866382	Rare genitourinary tumours	Recruiting	Phase 2	Interventional
Cabozantinib (AXL inhibitor)	NCT05136196	Melanoma or head and neck cancer	Recruiting	Phase 2	Interventional
Cabozantinib (AXL inhibitor)	NCT04551430	Metastatic soft tissue sarcoma	Active, not recruiting	Phase 2	Interventional
Cabozantinib (AXL inhibitor)	NCT03914300	Advanced differentiated thyroid cancer	Active, not recruiting	Phase 2	Interventional
Cabozantinib (AXL inhibitor)	NCT04079712	Poorly differentiated Neuroendocrine tumours	Active, not recruiting	Phase 2	Interventional
Cabozantinib (AXL inhibitor)	NCT04413123	Non‐clear cell renal cell carcinoma	Active, not recruiting	Phase 2	Interventional
Cabozantinib (AXL inhibitor)	NCT06132945	Renal cell carcinoma with brain metastases	Recruiting	Phase 1	Interventional
Cabozantinib (AXL inhibitor)	NCT05904080	Recurrent/metastatic nasopharyngeal cancer	Recruiting	Phase 2	Interventional
Cabozantinib (AXL inhibitor)	NCT05836571	Advanced soft tissue sarcoma	Active, not recruiting	Phase 2	Interventional
ONO‐7475 ± ONO‐4538	NCT03730337	Advanced or metastatic solid tumours	Completed	Phase 1	Interventional
ONO‐7475 ± Venetoclax	NCT03176277	Acute leukaemias/MDS	Terminated	Phase 1/2	Interventional
ONO‐7475 + Osimertinib	NCT06525246	EGFR‐mutant NSCLC	Active, not recruiting	Phase 1	Interventional
ONO‐7475 + ONO‐4538 + GnP/ONO‐7475 + GnP	NCT06532331	Metastatic pancreatic cancer	Active, not recruiting	Phase 1	Interventional
AVB‐S6‐500 + Avelumab	NCT04004442	Advanced urothelial carcinoma	Active, not recruiting	Phase 1	Interventional
AVB‐S6‐500 + PLD/Paclitaxel	NCT03639246	Platinum‐resistant recurrent ovarian cancer	Completed	Phase 1	Interventional
AVB‐S6‐500 + Paclitaxel/Carboplatin	NCT03607955	Ovarian, peritoneal or fallopian tube cancer	Withdrawn	Phase 1	Interventional
Batiraxcept (AVB‐S6‐500) + Paclitaxel	NCT04729608	Platinum‐resistant recurrent ovarian cancer	Terminated	Phase 3	Interventional
Batiraxcept (AVB‐S6‐500) + Nab‐paclitaxel/Gemcitabine	NCT04983407	Advanced pancreatic adenocarcinoma	Terminated	Phase 1/2	Interventional
AVB‐S6‐500 ± Cabozantinib ± Nivolumab	NCT04300140	Advanced/metastatic clear cell RCC	Terminated	Phase 1/2	Interventional
AVB‐S6‐500 + Durvalumab	NCT04019288	Platinum‐resistant/recurrent gynaecologic cancers	Terminated	Phase 1/2	Interventional
AVB‐500 (Batiraxcept) + Paclitaxel	NCT05826015	Recurrent high‐grade uterine cancer	Withdrawn	Phase 1	Interventional
MRX‐2843	NCT03510104	Advanced/metastatic solid tumours	Active, not recruiting	Phase 1	Interventional
MRX‐2843	NCT04946890	Relapsed/refractory acute myeloid leukaemia	Unknown	Phase 1/2	Interventional
MRX‐2843	NCT04872478	Relapsed/refractory AML, ALL or MPAL	Recruiting	Phase 1	Interventional
MRX‐2843 + Osimertinib	NCT04762199	Advanced EGFR‐mutant NSCLC	Recruiting	Phase 1	Interventional

### 5.4 Targeting ‘don't‐eat‐me’ signals

The ‘don't‐eat‐me’ stage represents one of the most direct strategies for re‐activating macrophage‐mediated anti‐tumour immunity by blocking inhibitory signals used by cancer cells to evade phagocytosis. This approach effectively ‘releases the brakes’ on macrophages, enabling them to attack otherwise protected malignant cells. The most prominent of these signals is CD47, an immune checkpoint protein overexpressed across various cancers that interacts with SIRPα on macrophages to transmit anti‐phagocytic signals. Beyond CD47, emerging signals such as CD24 are being explored as complementary targets.

#### 5.4.1 The CD47–SIRPα axis

Targeting the CD47–SIRPα axis aims to initiate the ‘pathological phagocytosis’ of live tumour cells rather than merely clearing apoptotic debris. Preclinical studies confirm that anti‐CD47 antibodies, such as Magrolimab, restore macrophage phagocytic function by disrupting this interaction.^230^ A pivotal strategy involves a ‘dual‐key’ synergistic effect, where CD47 blockade is combined with therapies that upregulate ‘eat‐me’ signals (e.g., CRT), such as chemotherapy, hypomethylating agents or BCL‐2 inhibitors like Venetoclax.^230^, ^231^, ^232^

First‐generation anti‐CD47 antibodies showed initial promise in early‐phase trials. Magrolimab combined with azacitidine achieved encouraging response rates in TP53‐mutant MDS and AML,^231^, ^233^ while its combination with rituximab demonstrated durable efficacy in refractory lymphomas.^234^, ^235^

However, the subsequent failure of two pivotal Phase III trials (ENHANCE‐2 and ENHANCE‐3) in AML to meet overall survival endpoints—accompanied by increased rates of fatal infections—provided critical lessons for the field:

1. Target‐related haematological toxicity: The broad expression of CD47 on red blood cells leads to dose‐limiting anaemia and haemagglutination, compromising patient safety and treatment intensity.^236^, ^237^
2. Lack of predictive biomarkers: Despite high response rates in TP53‐mutant cohorts, the lack of benefit in overall survival suggests a need for more refined biomarkers, such as TAM infiltration density or spatial CD47 distribution, to select responsive subpopulations.^238^

Optimisation of combinatorial strategies: The increased infection risk in ENHANCE‐3 suggests that complex multi‐drug regimens may exacerbate myelosuppression, necessitating more cautious dose‐scheduling and tumour‐specific trial designs.^237^

To overcome these hurdles, next‐generation CD47‐targeted agents leverage protein engineering to enhance safety. Ligufalimab utilises an IgG4 isotype to minimise Fc‐mediated effects on erythrocytes^239^; Evorpacept is a high‐affinity SIRPα‐Fc fusion protein with an inactivated Fc domain to reduce off‐target toxicity^240^, ^241^; and the bispecific antibody HX009 is engineered for reduced CD47 affinity to minimise RBC binding while retaining potent PD‐1 blockade.^242^ These advancements aim to preserve anti‐tumour activity while safeguarding erythrocyte homeostasis through precision engineering and biomarker‐driven stratification.

#### 5.4.2 The emerging CD24–Siglec‐10 axis

The CD24–Siglec‐10 axis has emerged as a novel ‘don't‐eat‐me’ pathway. The anti‐CD24 antibody IMM47 relieves the inhibition of live tumour cell phagocytosis and facilitates cell killing via ADCC, ADCP and CDC, showing synergy with PD‐1 inhibitors in preclinical models.^243^ Lessons from the CD47 field suggest that future clinical translation of CD24‐targeted agents must focus on its unique toxicity profile—given its expression on normal immune cells—and the development of CD24‐based patient stratification strategies and rational combination regimens.^243^

In summary, targeting ‘don't‐eat‐me’ signals is a validated strategy for unleashing innate anti‐tumour immunity. The evolution from first‐generation antibodies like Magrolimab to engineered, safety‐optimised agents such as Ligufalimab, Evorpacept and HX009 illustrates the field's iterative progress towards precision immunotherapy. By integrating deeper insights into the TME and developing robust biomarkers, these strategies are poised to convert the ‘silent evasion’ of cancer cells into a potent trigger for durable anti‐tumour responses (Table 3).

**TABLE 3:** Representative ‘don't‐eat‐me’–signal inhibitors, including CD47 and CD24 blockade agents, with mechanisms of action, preclinical evidence and clinical‐trial progress.

Drug name/target	NCT number	Tumour type treated	Status	Phases	Study type
HX009 (anti‐CD47/PD‐1 bispecific)	NCT05731752	Advanced solid tumours	Active, not recruiting	Phase 1	Interventional
HX009 (anti‐CD47/PD‐1 bispecific)	NCT04097769	Advanced solid tumours	Completed	Phase 1	Interventional
HX009 (anti‐CD47/PD‐1 bispecific)	NCT04886271	Advanced solid tumours	Unknown	Phase 2	Interventional
HX009 (anti‐CD47/PD‐1 bispecific)	NCT06708663	Biliary tract cancer, melanoma	Recruiting	Phase 1/2	Interventional
HX009 (anti‐CD47/PD‐1 bispecific)	NCT05189093	Relapsed/refractory lymphoma	Recruiting	Phase 1/2	Interventional
Lemzoparlimab (anti‐CD47)	NCT04912063	Acute myeloid leukaemia, myelodysplastic syndrome	Terminated	Phase 1	Interventional
Lemzoparlimab (anti‐CD47)	NCT04895410	Multiple myeloma	Terminated	Phase 1	Interventional
AO‐176 (anti‐CD47)	NCT03834948	Solid tumours	Completed	Phase 1/2	Interventional
AO‐176 (anti‐CD47)	NCT04445701	Multiple myeloma	Completed	Phase 1/2	Interventional
Evorpacept (CD47 inhibitor)	NCT05027139	HER2‐expressing cancers	Active, not recruiting	Phase 1/2	Interventional
Evorpacept (CD47 inhibitor)	NCT05787639	Oropharynx cancer	Recruiting	Phase 2	Interventional
Evorpacept (CD47 inhibitor)	NCT04675294	Head and neck squamous cell carcinoma	Active, not recruiting	Phase 2	Interventional
Evorpacept (CD47 inhibitor)	NCT05167409	Metastatic colorectal cancer	Active, not recruiting	Phase 2	Interventional
Evorpacept (CD47 inhibitor)	NCT05524545	Urothelial carcinoma	Completed	Phase 1	Interventional
Evorpacept (CD47 inhibitor)	NCT04755244	Acute myeloid leukaemia	Terminated	Phase 1	Interventional
Evorpacept (CD47 inhibitor)	NCT05002127	Gastric/GEJ adenocarcinoma	Active, not recruiting	Phase 2/3	Interventional
Evorpacept (CD47 inhibitor)	NCT07007559	Metastatic breast cancer	Recruiting	Phase 1/2	Interventional
Evorpacept (CD47 inhibitor)	NCT03013218	Advanced solid tumours and lymphoma	Completed	Phase 1	Interventional
Evorpacept (CD47 inhibitor)	NCT04675333	Head and neck squamous cell carcinoma	Active, not recruiting	Phase 2	Interventional
Evorpacept (CD47 inhibitor)	NCT05868226	Breast cancer, solid tumours	Recruiting	Phase 1	Interventional
Evorpacept (CD47 inhibitor)	NCT05467670	Ovarian cancer	Recruiting	Phase 2	Interventional
AK117 (anti‐CD47)	NCT04728334	Advanced solid tumours, lymphomas	Completed	Phase 1	Interventional
AK117 (anti‐CD47)	NCT05214482	Advanced malignant tumours	Active, not recruiting	Phase 1/2	Interventional
AK117 (anti‐CD47)	NCT04349969	Advanced solid tumours	Completed	Phase 1	Interventional
AK117 (anti‐CD47)	NCT05229497	Advanced malignant tumours	Unknown	Phase 1/2	Interventional
AK117 (anti‐CD47)	NCT04980885	Acute myeloid leukaemia	Active, not recruiting	Phase 1/2	Interventional
AK117 (anti‐CD47)	NCT06508606	Head and neck squamous cell carcinoma	Not yet recruiting	Phase 2	Interventional
AK117 (anti‐CD47)	NCT05382442	Metastatic colorectal cancer	Recruiting	Phase 2	Interventional
AK117 (anti‐CD47)	NCT05235542	Advanced malignant tumours	Unknown	Phase 1/2	Interventional
AK117 (anti‐CD47)	NCT06953999	Pancreatic cancer	Not yet recruiting	Phase 3	Interventional
AK117 (anti‐CD47)	NCT05227664	Triple‐negative breast cancer	Recruiting	Phase 2	Interventional
AK117 (anti‐CD47)	NCT06387420	Acute myeloid leukaemia	Not yet recruiting	Phase 1/2	Interventional
AK117 (anti‐CD47)	NCT06789848	Hepatocellular carcinoma, biliary tract cancer	Recruiting	Phase 2	Interventional
AK117 (anti‐CD47)	NCT06601335	Head and neck squamous cell carcinoma	Recruiting	Phase 3	Interventional
Magrolimab (anti‐CD47)	NCT03248479	Haematological malignancies	Terminated	Phase 1	Interventional
Magrolimab (anti‐CD47)	NCT05169944	Malignant brain tumours	Completed	Phase 1	Interventional
Magrolimab (anti‐CD47)	NCT04827576	Solid tumours	Terminated	Phase 2	Interventional
Magrolimab (anti‐CD47)	NCT02953782	Solid tumours, colorectal cancer	Completed	Phase 1/2	Interventional
Magrolimab (anti‐CD47)	NCT04778410	Myeloid malignancies	Terminated	Phase 2	Interventional
Magrolimab (anti‐CD47)	NCT03558139	Ovarian cancer	Completed	Phase 1	Interventional
Magrolimab (anti‐CD47)	NCT05829434	Acute myeloid leukaemia, MDS	Withdrawn	Phase 2	Interventional
Magrolimab (anti‐CD47)	NCT05627466	Acute myeloid leukaemia	No longer available	N/A	Expanded Access
Magrolimab (anti‐CD47)	NCT05330429	Metastatic colorectal cancer	Terminated	Phase 2	Interventional
Magrolimab (anti‐CD47)	NCT05367401	Myelodysplastic syndromes, AML	Withdrawn	Phase 1/2	Interventional
Magrolimab (anti‐CD47)	NCT04435691	Acute myeloid leukaemia	Terminated	Phase 1/2	Interventional
Magrolimab (anti‐CD47)	NCT04788043	Hodgkin lymphoma	Active, not recruiting	Phase 2	Interventional
Magrolimab (anti‐CD47)	NCT06046482	Head and neck squamous cell carcinoma	Terminated	Phase 2	Interventional
Magrolimab (anti‐CD47)	NCT05079230	Acute myeloid leukaemia	Terminated	Phase 3	Interventional
Magrolimab (anti‐CD47)	NCT04958785	Triple‐negative breast cancer	Terminated	Phase 2	Interventional
Magrolimab (anti‐CD47)	NCT02953509	Non‐Hodgkin lymphoma	Terminated	Phase 1/2	Interventional
Magrolimab (anti‐CD47)	NCT04892446	Multiple myeloma	Terminated	Phase 2	Interventional
Magrolimab (anti‐CD47)	NCT05929716	Large B‐cell lymphoma	Withdrawn	Phase 2	Interventional
Magrolimab (anti‐CD47)	NCT02216409	Solid tumours	Completed	Phase 1	Interventional
Magrolimab (anti‐CD47)	NCT05807126	Breast cancer, prostate cancer	Withdrawn	Phase 1	Interventional
Magrolimab (anti‐CD47)	NCT04854499	Head and neck squamous cell carcinoma	Terminated	Phase 2	Interventional
Magrolimab (anti‐CD47)	NCT02678338	Acute myeloid leukaemia, MDS	Completed	Phase 1	Interventional
Magrolimab (anti‐CD47)	NCT04599634	B‐cell lymphoma	Completed	Phase 1	Interventional
Magrolimab (anti‐CD47)	NCT04751383	Neuroblastoma, osteosarcoma	Completed	Phase 1	Interventional
Magrolimab (anti‐CD47)	NCT04778397	Acute myeloid leukaemia	Terminated	Phase 3	Interventional
Magrolimab (anti‐CD47)	NCT03869190	Urothelial carcinoma	Active, not recruiting	Phase 1/2	Interventional
Magrolimab (anti‐CD47)	NCT04541017	T‐cell lymphoma	Terminated	Phase 1/2	Interventional
Magrolimab (anti‐CD47)	NCT05823480	Acute myeloid leukaemia, MDS	Withdrawn	Phase 1	Interventional
Magrolimab (anti‐CD47)	NCT03922477	Acute myeloid leukaemia	Terminated	Phase 1	Interventional
Magrolimab (anti‐CD47)	NCT03527147	Non‐Hodgkin lymphoma	Completed	Phase 1	Interventional

### 5.5 Emerging frontiers: reshaping the metabolic and immunologic landscape of efferocytosis

Beyond direct targeting of ‘eat‐me’ or ‘don't‐eat‐me’ signals, the current research frontier focuses on reshaping the efferocytic process at the level of metabolic and immunologic programming.^244^ Rather than simply modulating individual engulfment events, these emerging strategies aim to fundamentally reprogram the functional output of macrophages, pivoting the TME from a suppressive state towards an immune‐active state.^245^

#### 5.5.1 Targeting metabolic sensing switches

The first strategy involves targeting metabolic sensors to reprogram the immune interpretation of phagocytes. The chloride‐sensing pathway has been identified as a critical metabolic switch determining the post‐efferocytic immune trajectory.^244^ Interfering with the key transporter SLC12A2 can enhance phagocytic efficiency while converting the outcome from immune silence to a pro‐inflammatory response, providing a novel target for reversing TAM‐mediated immunosuppression.^246^

#### 5.5.2 Intervening in post‐phagocytic processing

The second strategy targets post‐engulfment processing to directly reverse immune tolerance through two primary paths:

1. Inhibiting LC3‐associated phagocytosis (LAP): Disrupting this specialised phagocytic form reprograms macrophages from ‘tolerance inducers’ to ‘immune activators’, subsequently triggering the STING‐Type I IFN pathway to stimulate anti‐tumour T cell responses.^245^
2. Sequential intervention with thymosin α1: This immunomodulator binds to externalised PS on apoptotic cells and is internalised, activating specific signalling pathways within macrophages to directly antagonise efferocytosis‐induced M2 polarisation.^247^

#### 5.5.3 Altering cargo nature: Converting death into immunogenic signals

This approach aims to fundamentally reverse the immunological outcome of efferocytosis by altering the properties of the ‘cargo’ (the dying tumour cells). The core lies in applying therapies that induce ICD. For instance, anthracycline‐based chemotherapies and radiotherapy induce ER stress and DNA damage, causing dying tumour cells to expose CRT and release DAMPs such as HMGB1 and ATP.^248^, ^249^ These DAMPs act as potent ‘danger signals’, transforming otherwise tolerogenic apoptotic cells into in situ vaccines that initiate robust adaptive anti‐tumour immunity.^250^

#### 5.5.4 Systemic intervention in the TAM metabolic reprogramming network

The TME establishes a malignant cycle composed of intertwined amino acid, lipid and carbohydrate metabolic pathways through persistent efferocytosis.^17^

1. Amino acid axis: The Arg1 inhibitor INCB001158 (CB‐1158) disrupts the core cycle driving M2 polarisation by blocking the conversion of arginine to polyamines.^251^ This agent has shown promise in reshaping the immune landscape and demonstrated acceptable safety in combination with the anti‐PD‐1 antibody Retifanlimab.^252^ Other candidates include the dual arginase inhibitor OATD‐02 and IDO1 inhibitors.^253^, ^254^, ^255^
2. Lipid and glucose axes: CPT1a inhibitors aim to cut off the energy and signalling sources driving M2 polarisation.^256^ Meanwhile, inhibitors targeting glycolytic enzymes or monocarboxylate transporters (MCTs) break the lactate‐mediated support for tumour growth.^257^ Furthermore, IRE1α inhibitors target the ER stress‐induced metabolic reprogramming under hypoxia to sensitise tumours to immunotherapy.^258^

##### Summary: The paradigm shift towards metabolic reprogramming

Targeting the metabolic reprogramming of TAMs represents a paradigm shift in cancer immunotherapy. Its core value lies in transcending the superficial blockade of signalling pathways to fundamentally reprogram TAM cell fate through the intervention of chloride sensing,^244^ LAP^245^ and systematic metabolic networks.^251^, ^256^, ^257^

While metabolic targets possess potential as monotherapies, they primarily function as ‘metabolic enablers’ in clinical practice. As exemplified by the clinical study of INCB001158 plus PD‐1 blockade,^252^ these strategies dismantle the metabolic foundation of immunosuppression, creating a synergistic effect with adaptive immune activators. Future advancements will depend on a deeper resolution of TAM metabolic heterogeneity and the design of optimised, biomarker‐driven sequential combination regimens (Table 4).

**TABLE 4:** Summary of representative therapeutic strategies and agents targeting efferocytosis in the tumour microenvironment (TME).

Target/pathway	Representative agent	Mechanism of action (MOA)	Development stage	Major challenges
Sphingosine‐1‐phosphate (S1P) signalling axis	Suramin	Broad‐spectrum antagonist; inhibits S1P receptors and multiple growth factor signals to interfere with macrophage recruitment.	Preclinical and select clinical studies	Broad toxicity profile; high therapeutic heterogeneity; requires precise patient stratification.
	Fingolimod (FTY720)	S1P receptor modulator; functional antagonist of S1PR1; disrupts macrophage recruitment and lymphocyte trafficking.	Approved for MS; preclinical/early clinical for cancer	Balancing anti‐tumour effects with potential immunosuppression; necessitates combination therapy.
	Sonepcizumab	Anti‐S1P monoclonal antibody; neutralises extracellular ligands to block downstream signalling.	Phase II clinical trials	Limited monotherapy efficacy; requires combination strategies and biomarker guidance.
Phosphatidylserine (PS)	Bavituximab	Anti‐PS antibody; induces antibody‐dependent cellular cytotoxicity (ADCC) against PS+ cells and reprograms TAMs towards M1 polarisation.	Phase III (primary endpoint not met)	Narrow therapeutic window; lack of predictive biomarkers; combination strategies require optimisation.
AXL receptor	Bemcentinib	Selective AXL inhibitor; blocks immunosuppressive signalling of the PS–Gas6–AXL axis and reprograms TAMs.	Preclinical and early clinical	Establishing AXL as a predictive biomarker; optimising synergy with immunotherapy.
	ONO‐7475, TP‐0903 and so forth	AXL/MERTK inhibitors; reverse chemoresistance and reprogram macrophage polarisation.	Preclinical and early clinical	Understanding stage‐specific effects on efferocytosis; avoiding disruption of immune homeostasis.
CD47–SIRPα axis	Magrolimab	Anti‐CD47 antibody; blocks ‘don't‐eat‐me’ signal to promote macrophage phagocytosis of live tumour cells.	Phase III (select trials terminated)	Dose‐limiting haematological toxicity (anaemia); requires precise biomarkers and optimised combination regimens.
	Ligufalimab, Evorpacept and so forth	Next‐generation agents; engineered (e.g., IgG4 isotype, inactive Fc) to minimise haematological toxicity.	Early clinical	Maintaining efficacy while optimising safety; exploring optimal combination partners.
CD24–Siglec‐10 axis	IMM47	Anti‐CD24 antibody; blocks emerging ‘don't‐eat‐me’ signals; promotes phagocytosis and multiple killing effects.	Preclinical	Assessing unique toxicity due to broad expression on normal immune cells; needs patient stratification.
Metabolic reprogramming	INCB001158 (Arg1 inhibitor)	Blocks arginine metabolism; disrupts the cycle driving M2 polarisation and sustained efferocytosis.	Phase Ib clinical trial	Validating efficacy in complex metabolic networks; determining optimal combination therapies.
	OATD‐02 (dual Arg inhibitor)	Targets intracellular arginases; restores L‐arginine levels in the TME.	Preclinical/early clinical	Overcoming metabolic redundancy within the TME.
Post‐phagocytic programming	Thymosin α1	Binds to PS for endocytosis; activates intracellular signalling to directly antagonise M2 polarisation.	Preclinical	Complex MOA; requires clarity on optimal dosing sequences and indications.
Chloride‐sensing pathway	(Targeting SLC12A2, etc.)	Interferes with metabolic switches determining post‐phagocytic immune interpretation (anti‐inflammatory to pro‐inflammatory).	Early Exploration	Novel target; drug development is in early stages.
LC3‐associated phagocytosis (LAP)	(Specific inhibitors)	Inhibits LAP; reprograms macrophages from tolerance to activation via STING pathway.	Preclinical	Challenges in achieving specific LAP inhibition without affecting canonical autophagy.

## 6 CONCLUSION AND FUTURE PERSPECTIVES

In summary, targeting the sequential stages of efferocytosis—the ‘find‐me’, ‘eat‐me’ and ‘don't‐eat‐me’ phases—offers a multi‐dimensional therapeutic framework for reshaping the TME and overcoming current treatment barriers.^214^ From broad‐spectrum agents like suramin^193^ and specific S1P axis modulators like fingolimod^201^ and sonepcizumab,^207^ to precision interventions targeting PS recognition (e.g., Bavituximab^211^), post‐phagocytic signalling (e.g., AXL/MERTK inhibitors^216^) and the direct activation of macrophages via CD47/CD24 blockade,^230^, ^243^ this field has rapidly evolved from proof‐of‐concept to clinical iteration. Most notably, emerging metabolic intervention strategies offer a fundamental breakthrough by reprogramming TAM metabolic sensing,^244^ inhibiting post‐phagocytic processing such as LAP^245^ and modulating systemic metabolic networks (e.g., arginase^251^ and FAO^256^). These approaches convert immunosuppressive efferocytosis into immunogenic responses, addressing the inherent complexity and resistance of the TME.

Looking forward, the translation of these strategies from preclinical potential to tangible clinical benefit will depend on four pivotal directions:

1. Precision and biomarker‐driven stratification: Overcoming the therapeutic heterogeneity and toxicity of current agents necessitates the development of robust predictive biomarkers.^238^ Future research must identify markers that accurately reflect target dependency—such as AXL/CD47 expression levels,^218^ metabolic signatures or specific immune microenvironment profiles—to achieve precise patient selection.
2. Rational combinations and synergistic optimisation: Monotherapy targeting a single pathway is often insufficient to reverse the complex immunosuppressive network of the TME. Success will largely depend on mechanistically driven combination strategies.^221^ This includes combining ‘find‐me’ signal inhibitors with ICIs to co‐regulate innate and adaptive immunity^259^; sequentially pairing PS‐targeting antibodies with ICD‐inducing therapies (chemo/radiotherapy)^209^, ^248^; and utilising metabolic interventions, such as arginase inhibitors, as ‘enabling platforms’ to reshape the TME for T‐cell‐activating therapies.^252^
3. Technological iteration and pharmaceutical engineering: Learning from the setbacks of first‐generation agents, the optimisation of the therapeutic window is essential. This involves advanced protein engineering (e.g., Fc‐region optimisation, bispecific antibodies^242^ and ligand‐trapping fusion proteins^240^) and novel modalities like ADCs^228^ to minimise off‐target toxicities, particularly haematological side effects.^236^
4. Mechanistic depth and novel target discovery: Continued exploration of the molecular intricacies governing efferocytosis and its metabolic reprogramming will unveil novel druggable targets, such as specific ion channels,^246^ epigenetic regulators or unique metabolic enzymes, providing fresh impetus for therapeutic development.

In conclusion, targeting efferocytosis is a dynamic and rapidly advancing frontier in oncology. By integrating precision medicine, innovative combination regimens, and continuous drug optimisation, these interventions are poised to systematically reprogram tumour cell death from a ‘silent event’ of immune evasion into a ‘trigger’^250^ for durable anti‐tumour immunity, offering new hope for cancer patients worldwide.

## AUTHOR CONTRIBUTIONS

Conceptualisation, Qianxi Yang and Qianlu Yang; writing—original draft preparation, Qianxi Yang, Qianlu Yang and Jie Yan; writing—review and editing, Qianxi Yang, Qianlu Yang and Jie Yan; visualisation, Qianxi Yang, Qianlu Yang and Jie Yan; supervision, Qianxi Yang; project administration, Qianxi Yang. All the authors have read and agreed to the published version of the manuscript.

## CONFLICT OF INTEREST STATEMENT

The authors declare no conflicts of interest.

## ETHICS STATEMENT

Not applicable.

## CONSENT FOR PUBLICATION

Not applicable.
